# Upregulation of Retinal Dehydrogenase 2 in Alternatively Activated Macrophages during Retinoid-dependent Type-2 Immunity to Helminth Infection in Mice

**DOI:** 10.1371/journal.ppat.1002883

**Published:** 2012-08-23

**Authors:** Mara J. Broadhurst, Jacqueline M. Leung, K. C. Lim, Natasha M. Girgis, Uma Mahesh Gundra, Padraic G. Fallon, Mary Premenko-Lanier, James H. McKerrow, Joseph M. McCune, P'ng Loke

**Affiliations:** 1 Division of Experimental Medicine, Department of Medicine, University of California San Francisco, San Francisco, California, United States of America; 2 Division of Medical Parasitology, Department of Microbiology, New York University Langone Medical Center, New York, New York, United States of America; 3 Center for Discovery and Innovation in Parasitic Diseases, Department of Pathology, University of California San Francisco, San Francisco, California, United States of America; 4 Institute of Molecular Medicine, School of Medicine, Trinity College, Dublin, Ireland; St. Jude's Childrens Hospital, United States of America

## Abstract

Although the vitamin A metabolite retinoic acid (RA) plays a critical role in immune function, RA synthesis during infection is poorly understood. Here, we show that retinal dehydrogenases (Raldh), required for the synthesis of RA, are induced during a retinoid-dependent type-2 immune response elicited by *Schistosoma mansoni* infection, but not during a retinoid-independent anti-viral immune response. Vitamin A deficient mice have a selective defect in T_H_2 responses to *S. mansoni*, but retained normal LCMV specific T_H_1 responses. A combination of *in situ* imaging, intra-vital imaging, and sort purification revealed that alternatively activated macrophages (AAMφ) express high levels of Raldh2 during *S. mansoni* infection. IL-4 induces Raldh2 expression in bone marrow-derived macrophages *in vitro* and peritoneal macrophages *in vivo*. Finally, *in vivo* derived AAMφ have an enhanced capacity to induce Foxp3 expression in CD4+ cells through an RA dependent mechanism, especially in combination with TGF-β. The regulation of Raldh enzymes during infection is pathogen specific and reflects differential requirements for RA during effector responses. Specifically, AAMφ are an inducible source of RA synthesis during helminth infections and T_H_2 responses that may be important in regulating immune responses.

## Author Summary

Vitamin A deficiency, a major global health concern, increases morbidity and death due to infectious diseases. For vitamin A to be utilized by the immune system, it must be metabolized into retinoic acid (RA), its active form. RA is a key determinant of T cell activity. However, its contribution to protective immunity during infection is poorly understood, as is the regulation of its synthesis in this context. We examined RA synthesis by immune cells responding to helminth infection and virus infection. While intestinal T cell responses were vitamin A-dependent during both infections, only T cell responses elicited by helminth infection were vitamin A-dependent in the liver. Consistent with this finding, the enzymes necessary for RA synthesis were expressed by inflammatory cells recruited to the liver during helminth, but not virus, infection. We identified alternatively-activated macrophages as a source of RA synthesis within immune cells responding to helminth infection and find that they can induce regulatory T cells. Our findings provide a better understanding of vitamin A utilization during infection and demonstrate that RA synthesis is an inducible component of protective immunity.

## Introduction

Vitamin A (retinol) is a critical factor in protective immunity, as evidenced by the increase in infectious disease morbidity and mortality associated with its deficiency in the diet [1]. The biological activity of vitamin A requires intracellular oxidation of retinol to retinoic acid (RA), a hormone-like metabolite that modulates the function of innate and adaptive immune cells [2], [3]. The rate-limiting step in RA synthesis is catalyzed by three major isoforms of retinal dehydrogenase (Raldh1-3), a family of tightly regulated enzymes [4]–[6]. Homeostatic Raldh expression in immune cells is well described in gut-associated lymphoid tissues (GALT) [7]–[11], where RA synthesis by antigen presenting cells (APCs) contributes to the recruitment and function of local lymphocyte populations. However, it remains unclear whether Raldh expression is an inducible component of effector immune responses during infection in other peripheral organs like the liver.

Elucidating the regulation of RA synthesis by inflammatory cells is critical for understanding the role of RA signaling in shaping immune responses *in vivo*. While basal RA signaling is required for general T cell activation [12], RA also acts in concert with other signals to mediate inflammatory [13], [14] and regulatory [8], [10], [15], [16] T cell functions. In the presence of IL-4, a critical mediator of type-2 inflammation, RA favors T-helper (T_H_)2 responses in murine [17], [18] and human [19] CD4^+^ T cells by enhancing the expression of GATA-3 and type-2 cytokines while inhibiting T-bet and IFNγ expression. Accordingly, vitamin A deficiency attenuates eosinophilia, IgE responses, and type-2 cytokine expression *in vivo* [20]–[22]. T_H_2 cells mediate protective immunity to helminth parasites that are common in regions of the world where vitamin A deficiency is prevalent [23], [24]. However, the importance of RA in the generation of T_H_2 responses during helminth infection is not well characterized and the population of cells responsible for RA synthesis in this setting has not been identified.

In this study, we sought to determine whether RA synthesis is a regulated component of immune responses during infection. Based on the existing evidence that RA promotes T_H_2 responses, we hypothesized that Raldh expression is induced in APCs that are activated during T_H_2 inflammation. To address this hypothesis, we evaluated RA signaling and Raldh expression in mice infected with the parasitic helminth, *Schistosoma mansoni*, an important human pathogen that provides a well-characterized model of T_H_2 inflammation. Deposition of *S. mansoni* eggs in the liver and intestine drives a type-2 granulomatous response characterized by T_H_2 cells, AAMφ, and eosinophils [25]. In parallel, and in a model of T_H_1 responses, we evaluated mice infected with lymphocytic choriomeningitis virus (LCMV). The broad tropism of LCMV allowed for the direct comparison of T_H_1- and T_H_2-polarized responses in the liver and intestine. Vitamin A deficient mice showed severely impaired T_H_2 but not T_H_1 responses in the liver, suggesting a role for RA synthesis during T_H_2 inflammation at this site. Raldh enzymes were highly expressed by AAMφ recruited to liver granulomas during *S. mansoni* infection, and Raldh2 expression in macrophages was induced by activation with IL-4 *in vitro* and *in vivo*. Thus, our findings demonstrate that helminth-elicited AAMφ are an inducible source of RA synthesis in the setting of retinoid-dependent T_H_2 inflammation and identify IL-4 activation as a selective mechanism for Raldh2 induction in these cells.

## Results

### Vitamin A is critical for liver T_H_2 responses during *S. mansoni* infection

To assess the role of RA synthesis during infection, we first determined whether *S. mansoni*- and LCMV-elicited T cell responses are dependent upon vitamin A. Mice were maintained on a vitamin A deficient (A−) or control (A+) diet beginning at day 10 of gestation. *S. mansoni*-infected mice were analyzed at week 7 post-infection, corresponding to the acute T_H_2 response that is elicited by egg deposition, while LCMV (Armstrong strain)-infected mice were analyzed at day 7 post-infection. Infections were timed such that all the mice were analyzed at 15 weeks of age. By this time, serum retinol levels in A- mice were reduced to ∼0.35 µM, a level defined by the World Health Organization as severe vitamin A deficiency [26].

**Figure 1:**
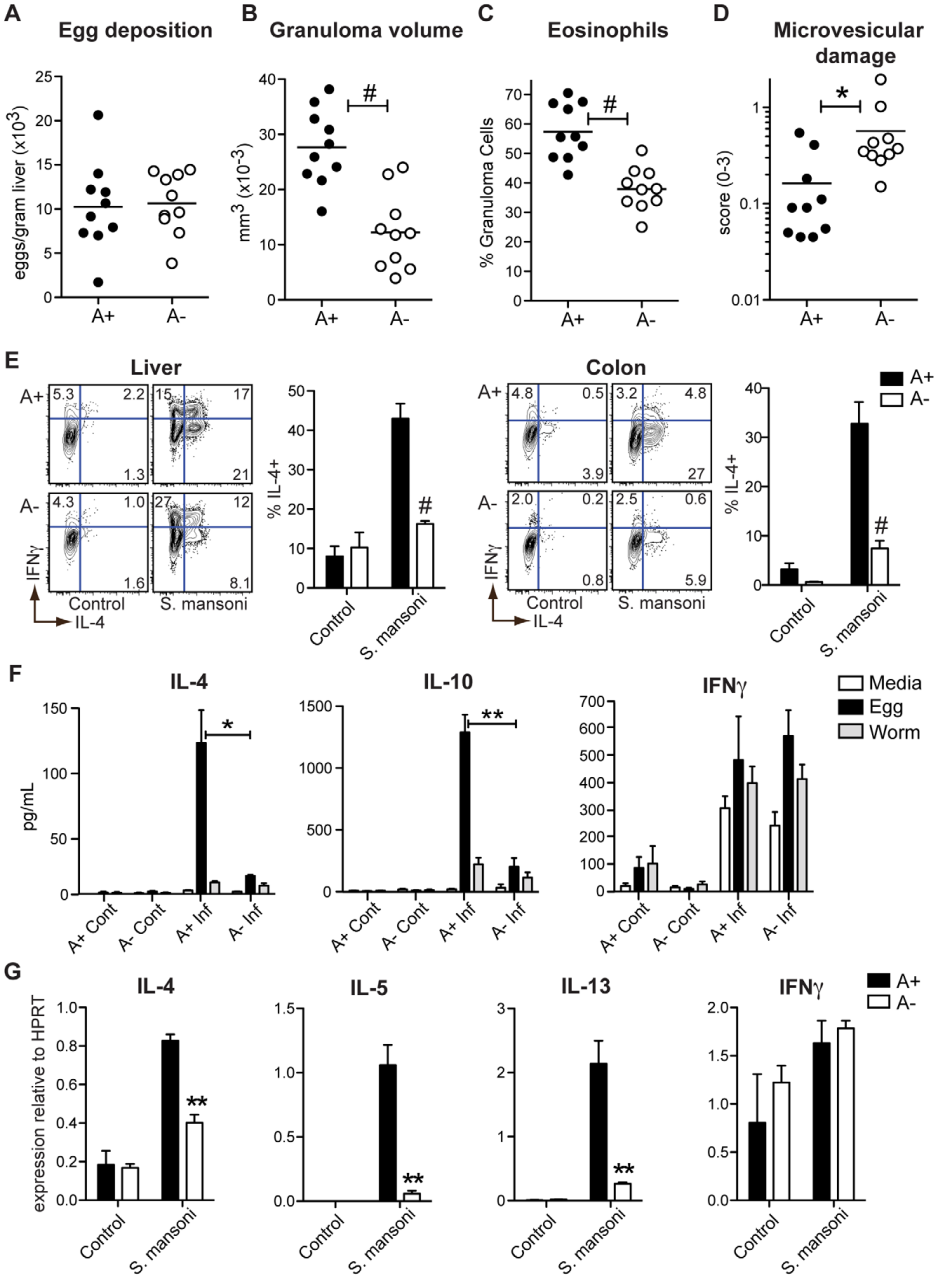
Vitamin A deficiency impairs *S. mansoni*-elicited Th2 responses. (**A**) Quantification of *S. mansoni* eggs deposited per gram of liver. (**B–D**) Histopathology of liver tissue sections stained with hemotoxylin and eosin and evaluated for granuloma volume (B), eosinophil infiltration (C), and microvesicular liver damage (D). (**E**) Flow cytometric analysis of intracellular cytokines expressed by cells harvested from the liver and colon following a 5-hour stimulation with PMA and ionomycin in the presence of brefeldin A. Representative contour plots are gated on live CD4^+^ T cells. n = 3–5 mice per group. (**F**) Cytometric bead array analysis of cytokine concentrations in culture supernatants. 3×10^5^ hepatic leukocytes harvested from *S. mansoni*-infected (Inf) and control (Cont) mice were cultured for 72 hours in the presence of egg homogenate (50 µg/mL), adult worm homogenate (50 µg/mL), or media alone. n = 3–4 mice per group. (**G**) qRT-PCR analysis of cytokine expression in hepatic leukocytes. Expression is normalized to HPRT. n = 3–5 mice per group. Error bars illustrate SEM; *p<0.05, **p<0.01, #p<0.001. Results are representative of two (A–D) or three (E, G) independent experiments.

Within the liver of *S. mansoni*-infected mice, eggs are deposited that evoke granulomatous eosinophilic inflammation, a process that is Th2-dependent. Although the livers of A+ and A− mice showed no differences in the numbers of eggs (Figure 1A), A− mice had significantly smaller granulomas (Figure 1B) and reduced eosinophilic infiltration (Figure 1C), similar to mice genetically deficient in T_H_2 responses (IL-4−/−, Stat6−/−) [27], [28]. The diminished granuloma size in A− mice was associated with microvesicular damage in the liver (Figure 1D). Unlike other models of more extreme liver pathology leading to mortality in *S. mansoni*-infected mice [27], [28], there was no difference in survival rates between A+ and A− mice.

**Figure 2:**
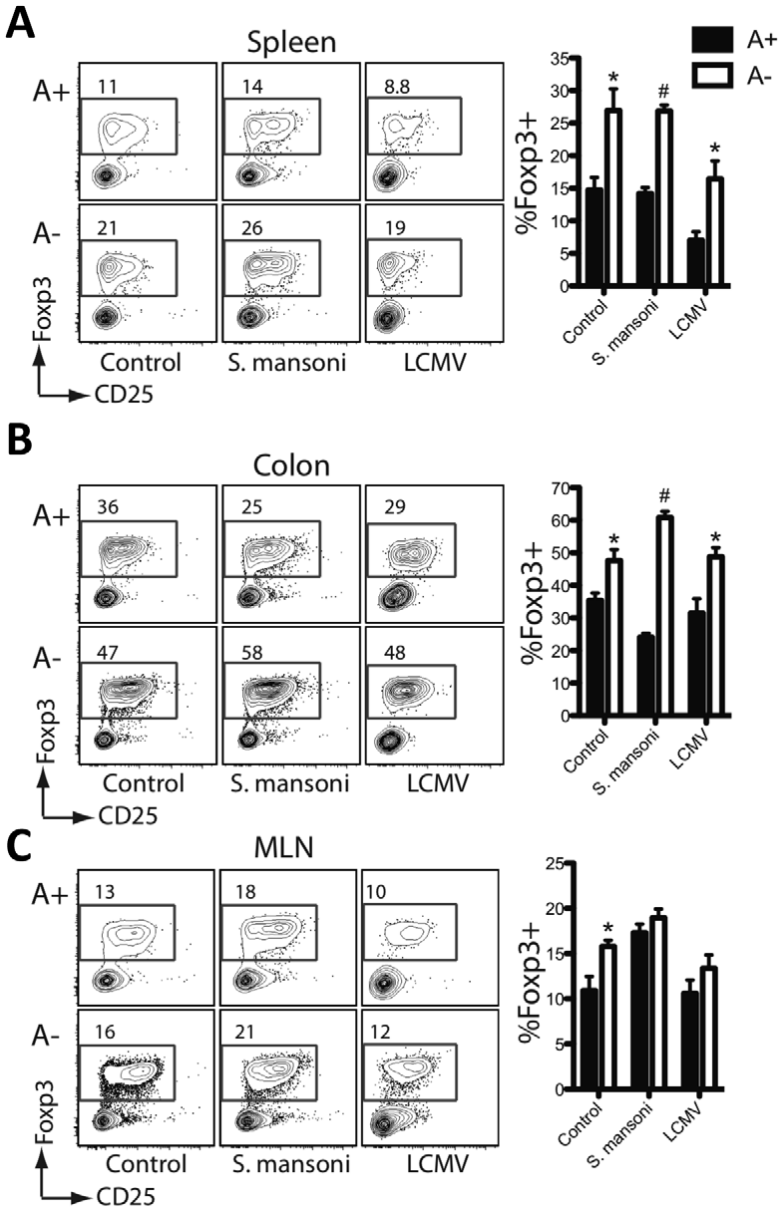
Foxp3^**+**^ regulatory T cells are increased during vitamin A deficiency. Flow cytometric analysis of intracellular nuclear Foxp3 staining on cells harvested from the liver (**A**), colon (**B**) and the mesenteric lymph nodes (**C**). Representative contour plots are gated on live CD4^+^ T cells. n = 3–5 mice per group. Error bars illustrate SEM; *p<0.05, #p<0.001. Results are representative of three independent experiments.

The characteristic expansion of IL-4^+^ T_H_2 cells associated with egg deposition in the liver and the intestine was significantly reduced in A− mice (Figure 1E). Concomitantly, the numbers of IFNγ^+^ and TNFα^+^ CD4^+^ T cells (Figures S1A and B) were not decreased in A− mice, indicating a selective defect in the T_H_2 response induced by vitamin A deficiency. Similar to reports in previous studies [12] Foxp3^+^ T cells were actually increased in the A− mice, either infected with *S. mansoni*, LCMV or even in the uninfected control mice (Figure 2 and S2). When hepatic leukocytes were co-cultured with schistosome egg antigen (SEA) for 72 hours, we found that SEA-specific IL-4 and IL-10 responses were dramatically reduced in A− mice. By contrast, the production of IFNγ and TNFα was indistinguishable between samples from A+ and A− mice (Figure 1F), although this response was not antigen specific and was probably not produced by CD4^+^ cells. By quantitative real-time PCR analysis (qRT-PCR) of isolated liver lymphocytes, we found that vitamin A deficiency significantly reduced the expression of IL-4, IL-5, and IL-13 but not of IFNγ (Figure 1G).

The effects of vitamin A deficiency were less pronounced in the draining mesenteric lymph nodes (MLN) than in the liver. Both the number of IL-4^+^ T cells analyzed e*x vivo* and the SEA-specific IL-4 and IL-10 responses were either unaffected or only slightly reduced by vitamin A deficiency (Figures S1C and D). However, the expression of IL-5 and IL-13 was vitamin A-dependent (Figure S1E). The majority of IL-4-producing T cells in lymph nodes responding to helminth infection are follicular helper-T cells (T-fh), which are functionally distinct from T_H_2 cells [29], [30]. In aggregate, these results suggest that RA signaling is critical for the expression of type-2 cytokines by T_H_2 cells recruited to sites of tissue inflammation, but is not essential for IL-4 expression by T-fh cells.

**Figure 3:**
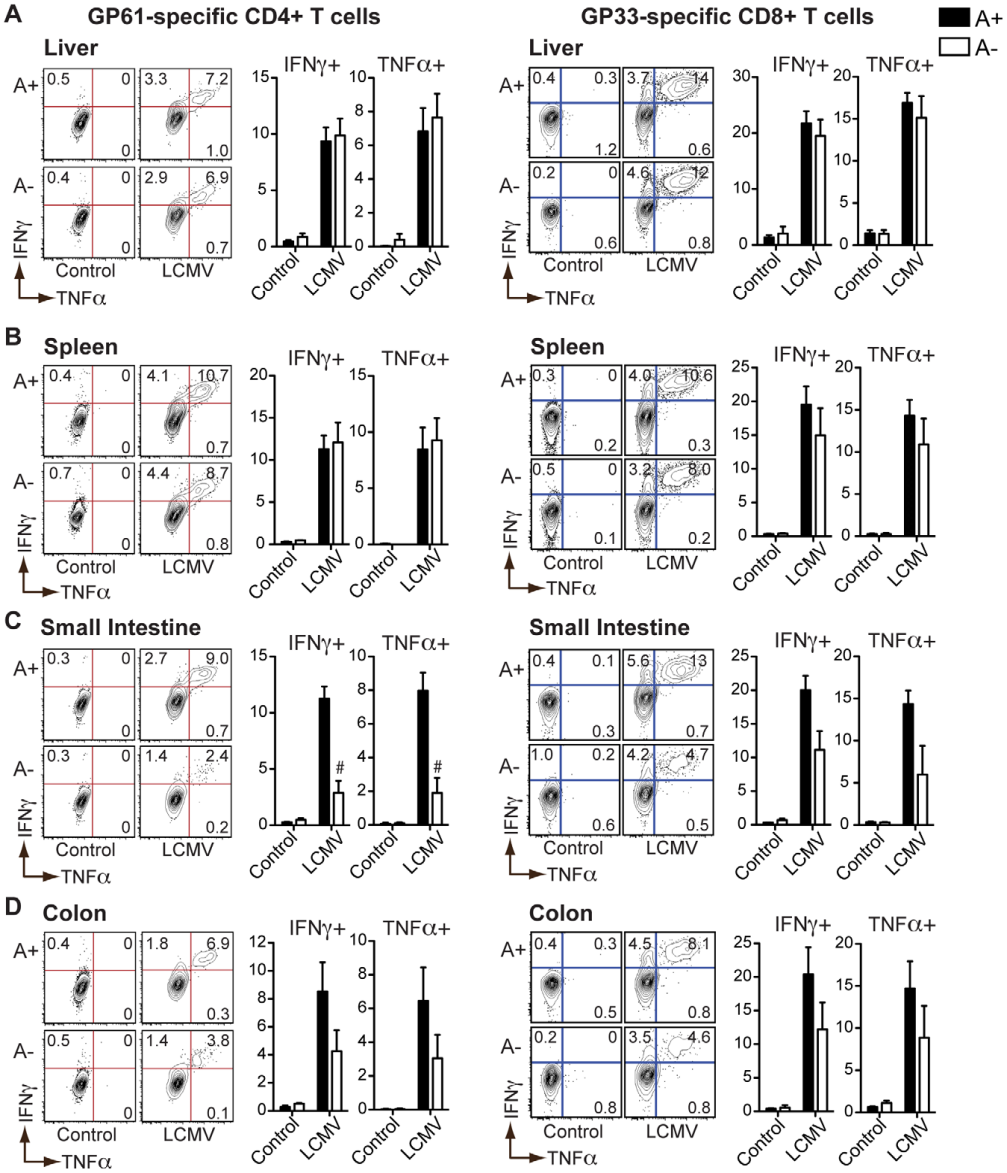
LCMV-specific T_H_1 responses in the intestinal mucosa are dependent on vitamin A metabolites. Flow cytometric analysis of intracellular cytokines expressed by cells harvested from the liver (**A**), spleen (**B**), small intestine (**C**), or colon (**D**) of LCMV-infected mice following a 5-hour stimulation with GP61 or GP33 peptides (10 µg/mL) in the presence of brefeldin A. Representative contour plots are gated on live CD4^+^ or CD8^+^ T cells. n = 3–5 mice per group. Error bars illustrate SEM; #p<0.001. Results are representative of three independent experiments.

In contrast to *S. mansoni* infection, we found that the numbers of GP61 and GP33 peptide-specific IFNγ- or TNFα-positive CD4^+^ or CD8^+^ T cells in the livers (Figure 3A), spleens (Figure 3B) and MLN (Figure S3) of LCMV-infected mice were unaffected by vitamin A deficiency. However, LCMV-specific (Figures 3C and D) as well as polyclonal (Figure S4) T_H_1 responses in the intestine were significantly diminished by vitamin A deficiency, consistent with a defect in intestinal homing [7].

These results demonstrate that vitamin A deficiency does not impair all T cell responses to pathogens, but that higher levels of RA signaling are required to maintain intestinal homing of effector T cells and to support helminth-elicited T_H_2 responses.

### *S. mansoni* infection induces systemic RA signaling in T cells

The vitamin A-dependency of *S. mansoni*-elicited T_H_2 responses suggested a critical role for RA during this infection. To determine whether RA signaling was directly targeted to CD4^+^ T cells during infection, we measured CCR9 expression by T cells as a surrogate marker of RA activity [7], [13].

**Figure 4:**
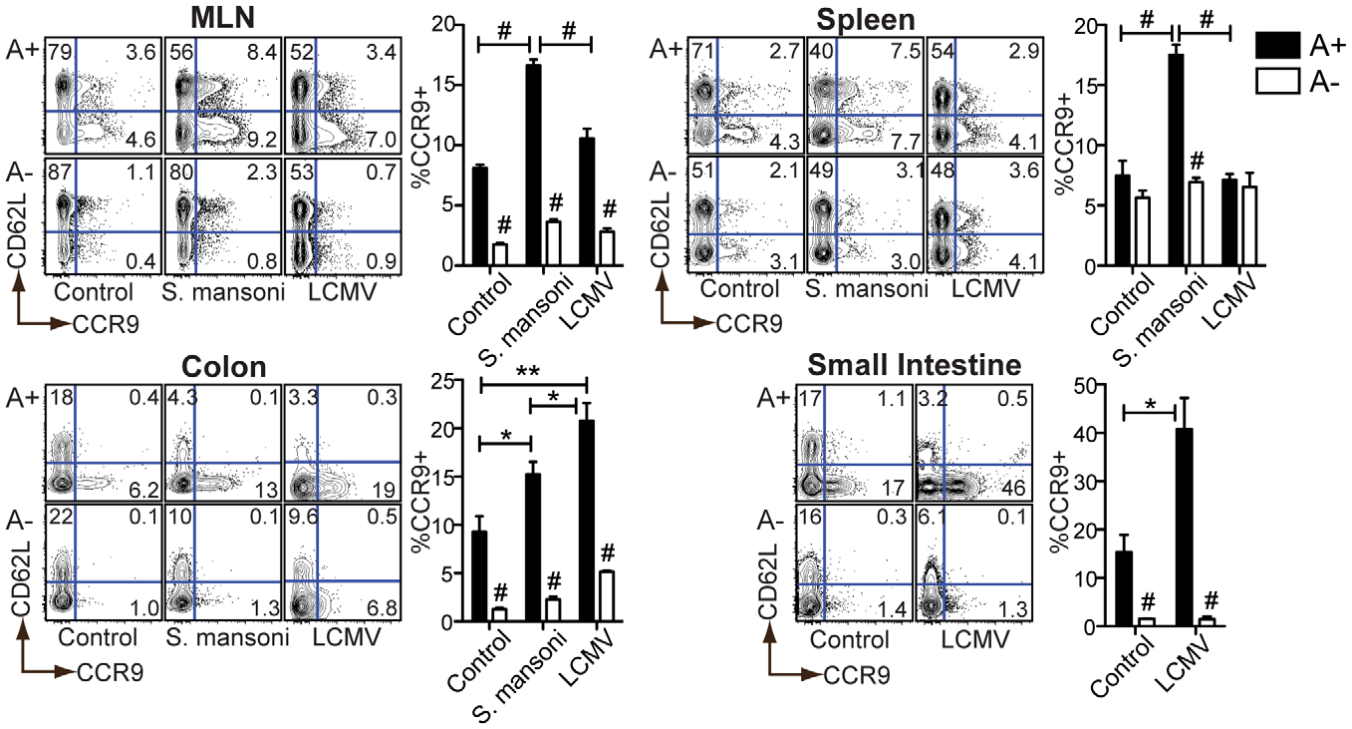
Retinoid-dependent CCR9 expression is variably induced during infection. Flow cytometric analysis of cells harvested at 7 weeks (*S. mansoni*) or 7 days (LCMV) post-infection (p.i.) from A+ or A− mice. Representative contour plots are gated on live CD4^+^ T cells. n = 3–5 mice per group. Error bars illustrate SEM; *p<0.05, **p<0.01, #p<0.001. Results are representative of three independent experiments. MLN = mesenteric lymph node.

Baseline CCR9 expression on CD4^+^ T cells in naïve, uninfected mice was reduced as a result of vitamin A deficiency in the MLN and intestinal mucosa but not in the spleen, confirming previous reports that homeostatic RA synthesis is a selective function of APCs in the GALT [7], [8], [10] (Figure 4). In the liver, CCR9 was not induced by either *S. mansoni* or LCMV infection, but was diminished in A− mice (Figure S5). As expected, all mucosal CCR9^+^ T cells were CD62L^neg^ (effector/memory subset), consistent with the possibility that these cells homed to the intestinal mucosa following antigen presentation.

During LCMV infection, CCR9 induction was restricted to the intestinal tissues. During *S. mansoni* infection, by contrast, CCR9 expression was also induced in secondary lymphoid organs (e.g., the spleen) (Figure 4). In each case, the increase in CCR9 expression was diminished in A− mice, indicating a dependency on vitamin A metabolites. These results indicate that *S. mansoni* infection requires vitamin A to drive RA signaling in T cells beyond the intestinal tissues.

### Type-2 inflammatory cells express RA-synthesizing enzymes

**Figure 5:**
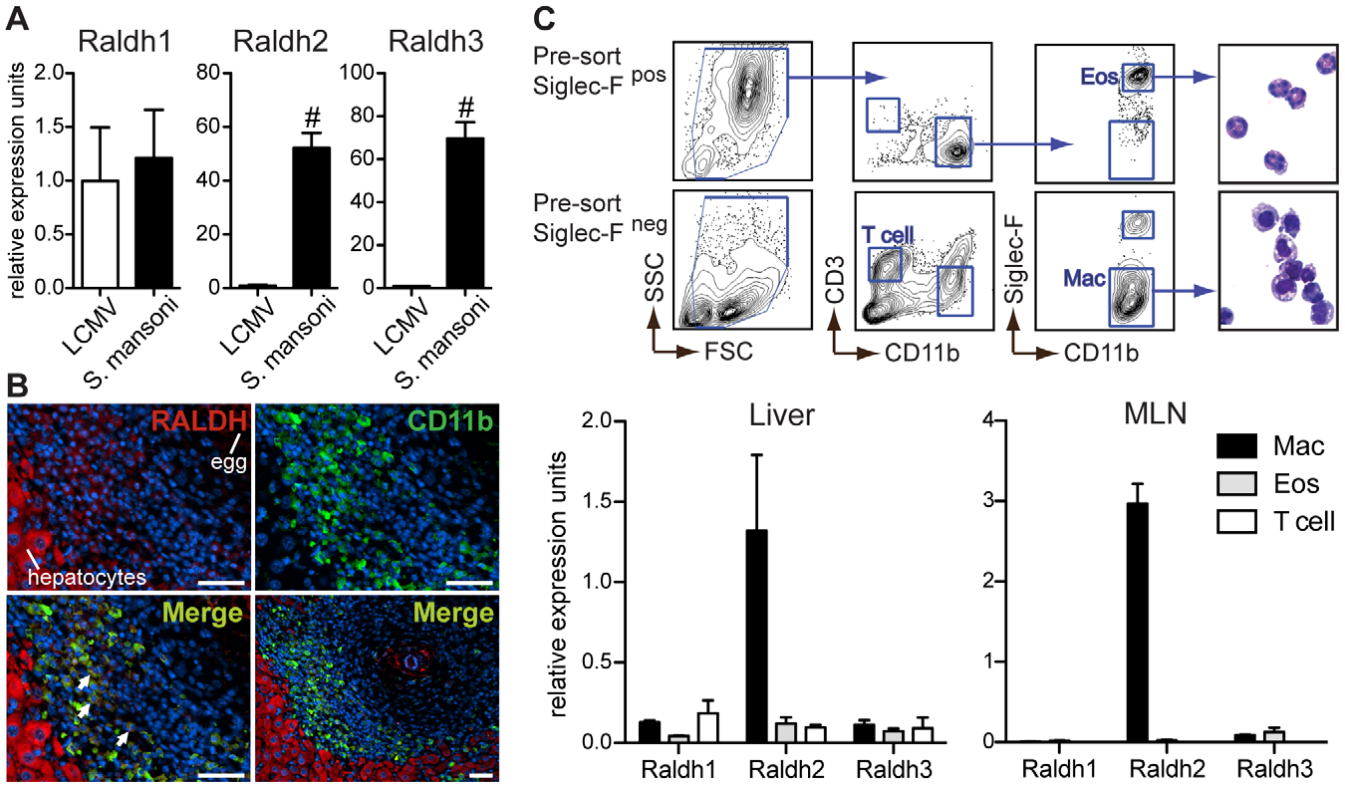
Type-2 inflammatory cells express Raldh2 and Raldh3, with Raldh2 most highly expressed in macrophages. (**A**) qRT-PCR analysis of retinal dehydrogenase (Raldh) expression (isoforms 1–3) in hepatic inflammatory infiltrates. Expression is normalized to HPRT and presented as fold-change above the average expression in LCMV samples. n = 3–5 mice per group. (**B**) Fluorescence microscopy of a hepatic granuloma co-stained with antibodies recognizing CD11b (green) and Raldh (red). Arrows point to cells within granulomas that co-stain for CD11b and Raldh. Scale bar = 50 µm. (**C**) qRT-PCR analysis of Raldh isoform expression in sorted macrophages (Mac), eosinophils (Eos), and T cells. Expression levels were normalized and presented relative to HPRT expression. Expression levels of Raldh isoforms are presented here on the same scale (different from panel A) in order to illustrate that Raldh2 is the most highly expressed isoform. Hepatic leukocytes from *S. mansoni*-infected mice were pre-sorted by microbead selection of Siglec-F^+^ cells. Cell fractions were then sorted to >90% purity by FACS, according to the gating strategy shown. A modified Giemsa stain demonstrated the expected morphology of sorted cells. n = 3 mice per group. Error bars illustrate SEM; #p<0.001. Results are representative of two (C) or three (A, B) independent experiments.

To determine which cells produce RA after infection, we used qRT-PCR to measure the three major Raldh isoforms that facilitate local RA synthesis in liver leukocytes isolated from *S. mansoni*- and LCMV-infected mice. Raldh2 and Raldh3 were expressed >50-fold higher in type-2 relative to type-1 inflammatory cells (Figure 5A), despite a similar increase in the number of inflammatory cells in the liver during both infections (data not shown). At day three post-infection with LCMV, when virus titers in the liver, intestine, spleen and MLN remain high, there was also no induction of any Raldh isoform expression in any of these tissues (data not shown). The MLN and spleen have a slightly higher expression of Raldh2 in *S. mansoni*-infected mice; however, these differences were slight and more variable compared to the liver (data not shown). No significant differences in Raldh2 expression were seen in the intestinal tissues of *S. mansoni*-infected mice (data not shown). Notably, Raldh2 is the isoform constitutively expressed by GALT APCs, while a role for Raldh3 in immunity has not been described.

*S. mansoni* egg-elicited granulomas are comprised of macrophages, eosinophils, and T cells [25]. To determine if myeloid cells are the source of Raldh expression, liver sections from *S. mansoni*-infected mice were co-stained with antibodies reactive for CD11b and Raldh. The Raldh antibody recognizes Raldh1 as well as Raldh2. Hepatocytes stained brightly for Raldh (Figure 5B), most likely reflecting expression of Raldh1, a low efficiency isoform highly expressed in the liver. Raldh staining was also detectable within granuloma cells that co-stained for CD11b. To distinguish between expression of different Raldh isoforms in macrophages and eosinophils, which both express CD11b, liver leukocytes from *S. mansoni*-infected mice were sort-purified by fluorescence activated cell sorting (FACS) for qRT-PCR analysis (Figure 5C). While expression of all three Raldh isoforms was detected in macrophages, eosinophils, and T cells, Raldh2 in macrophages was the most abundant source of Raldh expression. Similar results were obtained from sorted MLN cells (Figure 5C). However, in this tissue the sorting strategy does not exclude CD11b^+^ dendritic cells (DCs).

### AAMφ macrophages recruited to liver granulomas highly express Raldh2

**Figure 6:**
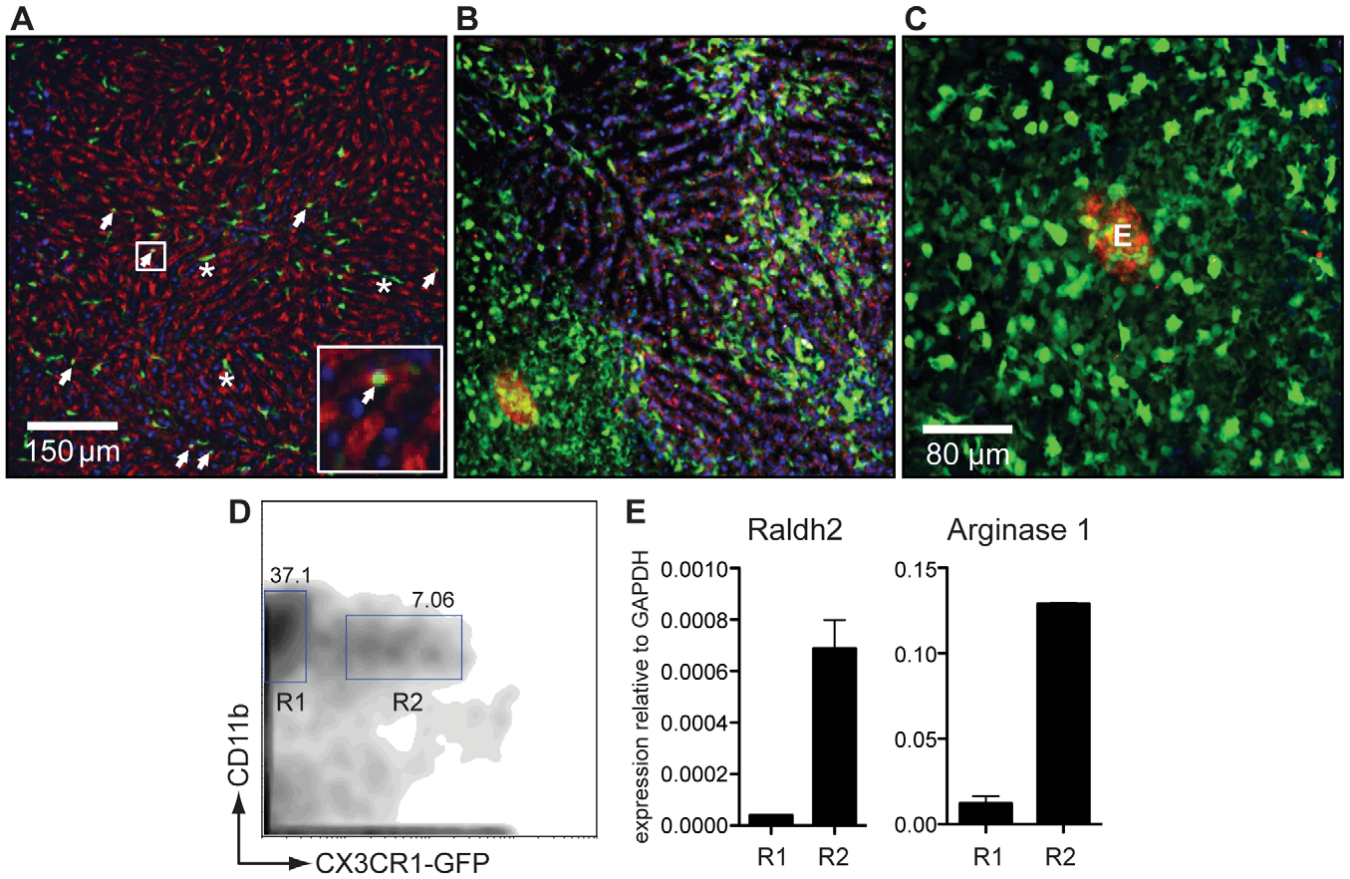
CX_3_CR1-GFP^+^ AAMφ in the liver granulomas of *S. mansoni* infected mice express Raldh2. (**A**) Intravital confocal image analysis of the liver of an uninfected CX_3_CR1-GFP/+ mouse showing that only round monocytes in the sinusoid vessels (white arrows), but not Kupffer cells, are GFP^+^. (**B**) The liver of an *S. mansoni*-infected mouse at seven weeks post infection [same magnification as (A)], showing that GFP^+^ cells predominate in the parenchymal tissue and not in the sinusoids, and have macrophage-like morphology with many cellular processes. (**C**) Confocal image analysis of a granuloma in the liver of a live, *S. mansoni*-infected CX_3_CR1-GFP/+ mouse. The autofluorescent egg can be seen in the red channel. (**D**) Flow cytometry sorting analysis of GFP^−^ (R1) and GFP^+^ (R2) CD11b^+^ cells isolated from the liver of an infected CX_3_CR1-GFP/+ mouse. (**E**) qRT-PCR analysis for arginase 1 and Raldh2 message performed on cDNA of FACS sorted cells shown in (E). Data are representative of three or more independent experiments.

Macrophages sorted from the whole livers of *S. mansoni*-infected mice may include inflammatory AAMφ recruited to granulomas as well as resident Kupffer cells. Recently, AAMφ have been reported to originate from the proliferation of tissue resident macrophages [31], which may also occur in the liver granulomas. The CX_3_CR1-GFP reporter mouse has been used to track monocyte-derived DCs and macrophages in several different organs, including the liver and the intestinal tract [32]. During steady state conditions, the only GFP^+^ cells were “round” monocytes (white arrows) in the sinusoidal vessels (Figure 6A) [32]. Kupffer cells did not express GFP, making this a convenient model to distinguish between them and inflammatory macrophages.

At seven weeks after infection with *S. mansoni*, almost all of the GFP^+^ cells found in the tissues had a morphology (with multiple cellular processes; Figures 6B and 6C) and a localization (on the outer fringe of granulomas; Figure 6C) consistent with that of AAMφ. To better define these cells, liver leukocytes were sort-purified into CD11b^+^ subpopulations that were either positive or negative for GFP (Figure 6D). RNA was extracted from these fractions and the expression of arginase 1, Ym1, FIZZ1 as well as of Raldh2 was measured by qRT-PCR analysis within them (Figure 6E). Compared to CD11b^+^CX_3_CR1-GFP^−^ cells, CD11b^+^ CX_3_CR1-GFP^+^ cells expressed high levels of arginase 1, Ym1, FIZZ1 and of Raldh2, indicating that CX_3_CR1-GFP^+^ cells are AAMφ and an important source of RA synthesis during *S. mansoni* infection.

### IL-4 activation induces Raldh2 expression in macrophages

**Figure 7:**
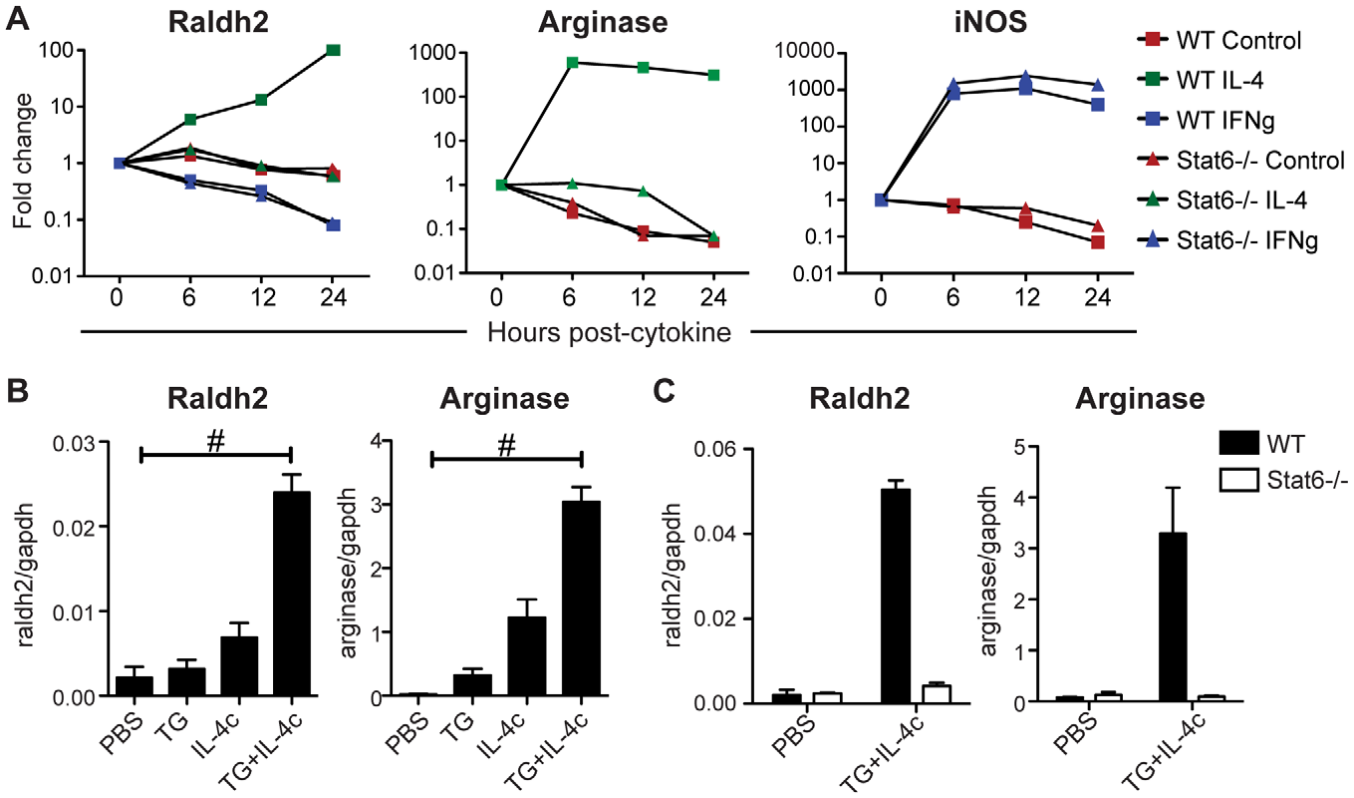
IL-4 induces Stat6-dependent Raldh2 expression in macrophages *in vitro* and *in vivo*. (**A**) qRT-PCR analysis of cytokine-treated bone marrow-derived macrophages from WT and Stat6^−/−^ mice. Expression is normalized to HPRT and presented as a fold-change above untreated cells. (**B, C**) qRT-PCR analysis of peritoneal macrophages elicited by i.p. administration of thioglycollate (TG) and/or IL-4 complexes (IL-4c) from WT and Stat6^−/−^ mice. Expression is normalized to GAPDH. n = 2–4 mice per group. Error bars illustrate SEM; #p<0.001. Results are representative of two (B) or three (A) independent experiments.

To further explore the regulation of Raldh expression by AAMφ, bone marrow-derived macrophages were treated with IL-4 or IFNγ *in vitro* and then assayed for expression of Raldh2 transcript using qRT-PCR. Stat6^−/−^ macrophages were activated in parallel to confirm the specificity of IL-4 signaling. As expected, IL-4-induced arginase 1 expression was strictly Stat6-dependent while IFNγ-induced iNOS expression was unaffected in Stat6^−/−^ macrophages (Figure 7A). Raldh2 showed Stat6-dependent induction by IL-4. By contrast, Raldh2 expression was inhibited by IFNγ and not affected by the regulatory cytokines, IL-10 and TGF-β1 (data not shown). We did not detect Raldh1 or Raldh3 expression in bone marrow-derived macrophages under any of these culture conditions. These results support the conclusion that Raldh2 expression is a selective characteristic of AAMφ and not of classically-activated macrophages.

Next, Raldh expression was assayed in AAMφ elicited *in vivo* by intraperitoneal administration of thioglycollate (TG) in combination with recombinant IL-4 complexed with anti-IL-4 antibodies (IL-4c) [31]. Raldh2, like arginase 1 and also Ym1 and FIZZ1 (Figure S6), was highly expressed in peritoneal macrophages elicited by TG plus IL-4c treatment compared to treatment with TG or IL-4c alone, or to resident peritoneal macrophages (PBS control; Figure 7B). Raldh2 induction by this method was abrogated in Stat6^−/−^ mice (Figure 7C).

### AAMφ can convert naïve CD4^+^ T cells into Foxp3^+^ T cells

**Figure 8:**
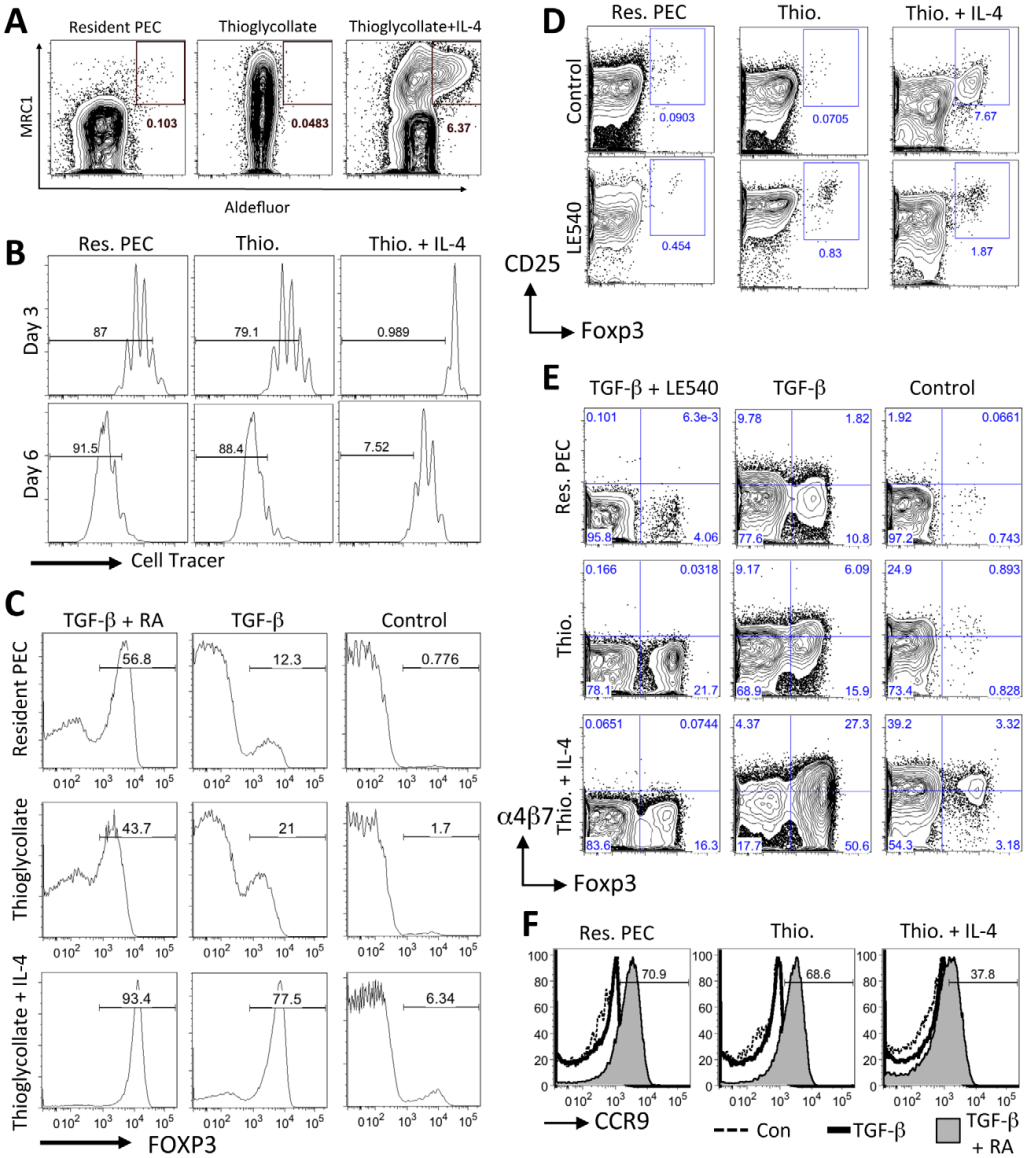
AAMφ have an enhanced capacity to induce Foxp3 expression in T cells. (**A**) Flow cytometric analysis of aldehyde dehydrogenase (ALDH) activity in peritoneal cells using the Aldefluor assay. Representative contour plots are gated on live F4/80^+^CD11b^+^ cells. (**B**) Flow cytometric analysis of activated (anti-CD3+IL-2) labeled naïve CD4^+^ cells (gated on CD11b^−^, CD3^+^, live Hoechst negative, CD4^+^ cells) cultured with peritoneal macrophages from control untreated mice (Res. PEC), mice injected with Thioglycollate (Thio.) or mice injected with Thioglycollate and IL-4 (Thio.+IL-4). Gates show the frequency of cells that have undergone more than 2 rounds of cell division (at Day 3) or more than 4 rounds of cell division (at Day 6). (**C**) Flow cytometry histogram plots showing the percentage of co-cultured CD4^+^ cells expressing Foxp3 (at Day 6) when activated with anti-CD3 and IL-2 alone (Control) or with exogenous TGF-β1 (2 ng/ml) or TGF-β1 and RA (100 nm). (**D**) Flow cytometry contour plots showing the percentage of CD25^+^, Foxp3^+^ CD4^+^ T cells after 6 days of co-culture with the RA inhibitor LE540 (1 µM). (**E**) Flow cytometry contour plots showing the effects of TGF-β1 and LE540 on the expression of α4β7 integrin and Foxp3 in co-cultured (Day 6) CD4^+^ cells. (F) Flow cytometry histogram plots showing the induction of CCR9 by TGF-β1 and RA (solid grey) when compared to CD4^+^ cells cultured with anti-CD3 and IL-2 alone (Control, dashed line) or with the addition of TGF-β1 only (solid thick line). Results are all representative of two or three independent experiments.

AAMφ elicited by TG plus IL-4c treatment were then assayed for aldehyde dehydrogenase (ALDH) activity by flow cytometry using the Aldefluor assay. Peritoneal F4/80^+^CD11b^+^ macrophages expressing the mannose receptor MRC1 had abundant ALDH activity that was blocked by the ALDH-specific enzyme inhibitor diethylaminobenzaldehyde (DEAB) (Figure 8A and S6). These results show that ALDH activity mirrors expression of Raldh2 in AAMφ and confirm that inflammatory AAMφ are an inducible source of RA synthesis. Inflammatory macrophages elicited by thioglycollate alone expressed MRC1, but did not have ALDH activity.

To determine if these AAMφ can induce Foxp3^+^ T cells, as has been previously demonstrated for RA-producing CD103^+^ lamina propria DCs [8], [10], naïve T cells were cultured with AAMφ elicited by TG plus IL-4c treatment, or with TG-elicited macrophages and resident peritoneal macrophages. As was shown previously for AAMφ elicited by *Brugia malayi* [33] and *S. mansoni* [34], AAMφ elicited by TG plus IL-4c significantly inhibited the proliferation of naïve CD4^+^ T cells (Figure 8B). By day 6 of co-culture, significantly more Foxp3^+^ CD4^+^ cells were detected after culture with AAMφ elicited by TG plus IL-4c (Figure 8C, right panel), relative to culture with resident macrophages or TG-elicited macrophages indicating that AAMφ have an enhanced capacity to induce Foxp3^+^ CD4^+^ cells.

TGF-β1 has been previously shown to enhance conversion of naïve CD4^+^ T cells into Foxp3^+^ cells, especially in the presence of CD103^+^ DCs. Addition of exogenous TGF-β1 to the cultures enhanced the induction of Foxp3^+^ CD4^+^ cells (Figure 8C) even with resident macrophages (12.3%) and TG-elicited macrophages (21%), but was especially dramatic with AAMφ elicited by TG plus IL-4 (77.5%). When exogenous TGF-β1 was supplemented with exogenous RA, there was an even greater induction of Foxp3^+^ cells. Almost all of the CD4^+^ cells were Foxp3^+^ in cultures with AAMφ, compared with approximately half of the CD4^+^ cells being Foxp3^+^ in cultures with resident macrophages or TG-elicited macrophages.

We then determined if the conversion of naive CD4^+^ T cells into Foxp3^+^ T cells by AAMφ could be inhibited by a synthetic RA receptor inhibitor LE540 (Figure 8D and 8E). Whereas LE540 slightly increased the proportion of CD4^+^ CD25^+^ Foxp3^+^ cells when added to cultures with resident peritoneal macrophages or TG-elicited macrophages, LE540 very significantly blocked the induction of CD25^+^ Foxp3^+^ cells by AAMφ (Figure 8D). When LE540 was added to the cultures along with TGF-β1 (Figure 8E), there was a reduction in the number of Foxp3^+^ cells induced by AAMφ to a similar level (16.3%) as TG-elicited macrophages (21.7%). There was also a reduction in the expression of α4β7 integrin, which was previously been shown to be induced by RA (Figure 8E). Notably, the induction of α4β7 integrin by AAMφ was also reduced in the presence of LE540. However, CCR9 was not induced by culture with AAMφ (Figure 8D) and even when RA was added exogenously to the cultures, fewer CD4^+^ cells (37.8%) were CCR9^+^ when cultured with AAMφ compared to resident macrophages (70.9%) or TG-elicited macrophages (68.6%). These results indicate that while AAMφ may induce Foxp3 expression through RA, they do not directly induce CCR9 expression on naïve CD4^+^ cells *in vitro* and may actually inhibit CCR9 expression.

## Discussion

The increase in infectious disease morbidity and mortality associated with vitamin A deficiency can be reduced by vitamin A supplementation, suggesting that vitamin A metabolites are important in reducing the pathogenic effects of infection [1], [2]. RA mediates the effects of vitamin A in adaptive immunity [7], [11], [12], [17]; however, the regulation of RA synthesis from retinoid precursors during infection remains poorly understood. In this study we show that AAMφ could be an important source of RA during a T_H_2 response against helminths.

This study was aimed at determining whether RA synthesis is an inducible function of pathogen-elicited immune responses. We first evaluated the dependency of effector immune responses on dietary retinoids and identified two critical roles for RA in regulating T cell responses during infection: (1) in the gut, where RA is constitutively synthesized by GALT APCs and drives intestinal homing, both T_H_2 and T_H_1 responses were retinoid-dependent; and (2) the induction of systemic RA signaling during helminth infection corresponded to the retinoid-dependency of T_H_2 but not T_H_1 responses in the liver. The latter finding suggested that RA synthesis might be a specialized function of type-2 inflammatory cells. Indeed, we found that infiltrating leukocytes in the liver during *S. mansoni* infection expressed high levels of the RA-synthesizing enzymes Raldh2 and Raldh3, with Raldh2 abundantly expressed by AAMφ that had been recruited to granulomas. AAMφ are a common feature of type-2 immune responses [35] and have been implicated in T cell regulation, fibrosis, and mucosal repair.

The regulation of RA synthesis in DCs is much better understood than in macrophages. While the signals in the gut microenvironment that drive constitutive RA synthesis are not well understood, IL-4 [36], GM-CSF [37], and beta-catenin signaling [38] have been implicated in Raldh2 expression by GALT DCs. TLR2 stimulation [39] and activation of the peroxisome proliferator-activated receptor (PPAR)-γ [40] can also induce Raldh2 expression by DCs. More recently, RA itself has also been shown to induce Raldh enzyme activity in DCs [37], [41]–[43]. Consistent with our finding that AAMφ represent an important Raldh2-expressing population during *S. mansoni* infection, we found that IL-4 drives Raldh2 expression in macrophages *in vitro* and *in vivo*. Raldh2 in macrophages appeared to be the dominant source of Raldh expression in type-2 inflammatory cells; however, the catalytic efficiency (V_max_/K_m_) of Raldh3 is ∼10-fold higher than Raldh2 [6]. It is accordingly possible that both of these enzymes are relevant sources of RA synthesis within *S. mansoni* granulomas. Raldh3 expression was nearly undetectable in the liver of uninfected and LCMV-infected mice, suggesting the specificity of this enzyme for type-2 inflammation. Further studies are needed to elucidate the signals mediating Raldh3 induction.

While this study focused on the role of RA signaling in T cell responses, the induction of RA synthesis during helminth infection has important implications for other cell types involved in type-2 inflammation. For example, RA promotes eosinophil survival by inhibiting caspase-3 expression and function [44]. RA also inhibits IL-12 expression in DCs [45] and macrophages [46], reducing the T_H_1-priming capacity of these cells. Interestingly, IL-3 activation has been shown to induce Raldh2 expression in human basophils *in vitro*, leading to both autocrine and paracrine RA signaling [47]. Further investigation into these RA-mediated effects *in vivo* may better define the role of vitamin A and AAMφ in protective immunity.

While we identified AAMφ to be an important source of Raldh2 activity (and hence a source of RA) during *S. mansoni* infection, we have not addressed the relative contribution of DC-derived RA in regulating T cells during infection. DCs can also become alternatively activated during helminth infection [48] and the RA produced by AAMφ may act directly on DCs to enhance Raldh enzyme activity [41]–[43] through a positive feedback loop mechanism. Since DCs are much better at presenting antigen to naïve T cells in draining lymph nodes, they may be more important for regulating T cell differentiation through RA than AAMφ. Instead, AAMφ may condition DCs to produce RA when they migrate to draining lymph nodes during infection. We found that AAMφ could not induce CCR9 expression on naïve T cells *in vitro*, suggesting that DCs may be more important in performing this function. The generation of mice with macrophage- and DC-specific defects in Raldh expression will be critical in further exploring the relative contribution of RA synthesis from these two APC populations during immune responses to infections. These experiments could also provide more direct evidence for the promotion of T_H_2 responses by RA produced by AAMφ or DCs.

RA promotes Foxp3^+^ regulatory T cell (Treg) induction *in vitro* [8], [10], [15], [16], and previous studies have highlighted the selective ability of Raldh2-expressing GALT DCs to induce Foxp3 expression in T cells in a RA-dependent manner [8], [10]. In this study, we made the important observation that AAMφ, like lamina propria CD103^+^ DCs [8], [10] can induce the differentiation of Foxp3^+^ T cells through an RA-dependent mechanism. While lamina propria macrophages have been described to induce Foxp3^+^ T cells [9], this is the first time that AAMφ have been shown to be a source of RA and have the capacity to induce the differentiation of Foxp3^+^ T cells. Since AAMφ and Foxp3^+^ T cells are both important in regulating the immune response during helminth infection [24] it is perhaps not a surprise that AAMφ can induce the differentiation of Foxp3^+^ T cells. Future studies will determine if our observations made through an *in vitro* system are indeed functionally relevant during a complex *in vivo* infection process.

It is unclear why vitamin A deficient mice have more Foxp3^+^ Tregs than mice on a control diet, either under baseline, uninfected conditions, or when infected with LCMV or *S. mansoni*. Other recent studies have also shown a higher frequency of lamina propria Tregs in vitamin A deficient mice and mice lacking RA receptor (RAR)-α [12], [49]. While the higher frequency of lamina propria Tregs observed in vitamin A deficient and RAR-α^−/−^ mice could be attributable to a loss of effector CD4^+^ T cells in this tissue rather than an increase in the number of Tregs [12], we also observed higher Treg frequencies in the MLN and spleen of vitamin A deficient mice. Notably, vitamin A deficiency had no effect on thymic Treg frequency (Figure S2). Further studies are needed to determine the mechanism of expansion and suppressive function of Foxp3^+^ Tregs induced during vitamin A deficiency.

Although it has previously been shown that T_H_1 and T_H_17 responses are attenuated in vitamin A deficient mice (e.g. during infection with *Toxoplasma gondii*) [3], [12], we find here that responses to LCMV are mostly intact, apart from the homing of activated T cells to the intestinal tissues. The predominantly CD8^+^ CTL response to LCMV may have different requirements for RA than intracellular parasite and bacterial pathogens that elicit T_H_1 responses. Future experiments with RAR-deficient mice may clarify the role of RA for CTL responses during viral infections such as LCMV.

Vitamin A deficiency affects ∼200 million preschool age children and ∼19 million pregnant woman globally [26], both of which populations are also at great risk for severe infections. The geographic distribution of vitamin A deficiency overlaps significantly with that of endemic helminth infections. We have demonstrated that RA-synthesizing enzymes are induced during retinoid-dependent type-2 immunity and our results support a role for RA in the generation of protective T_H_2 responses during helminth infection. Importantly, Raldh2 expression was found to be a selective function of AAMφ, an APC population that is common to a variety of helminth infections [50], [51] and required for host protection during schistosomiasis [52]. It follows that the efficacy of vaccines aimed at eliciting protective T_H_2 responses against helminth parasites [53] may depend on both the vitamin A status of the host as well as on the ability to prime APCs such as AAMφ that are competent for RA synthesis.

## Materials and Methods

### Mice

Wild-type and Stat6^−/−^ C57BL/6 mice were purchased from Jackson Laboratories. CX_3_CR1-GFP mice were kindly provided by Dr. Dan Littman (Skirball Institute, NYU) and were used as heterozygotes from crosses of CX3-CR1-GFP/GFP with wild-type C57BL/6 mice. For vitamin A deficiency experiments, timed-pregnant C57BL/6 dams were purchased from Charles River. Mice were maintained in a specific pathogen free UCSF Laboratory Animal Resource Center facility. Pregnant dams were fed a vitamin A deficient (0 IU/g, TD.86143 Harlan Teklad) or control (20,000 IU/g, TD.93160) diet starting at day 10 of gestation and continuing through weaning. After weaning, mice were maintained on the same diet for the duration of the experiment. Animal protocols were approved by the UCSF Institutional Animal Care and Use Committee.

### Infections

Mice were infected subcutaneously with 150 Puerto Rican *S. mansoni* cerceriae harvested from laboratory-maintained *Biomphalaria glabrata* snails. This number was titrated to result in a consistent chronic non-lethal infection in C57BL/6 mice. The intensity of infection was determined by counting adult worms recovered by perfusion of the portal system at euthanasia. To determine hepatic egg burden, liver samples were weighed, homogenized, and digested with trypsin; eggs were then sedimented and counted under a dissecting microscope. 2×10^5^ p.f.u. of LCMV-Armstrong was administered intraperitoneally.

### Treatment with IL-4 complexes (IL4c)

Mice were treated i.p. on day 0 and day 2 with IL-4c mixture containing 5 µg of recombinant murine IL-4 (Peprotech) and 25 µg of anti-IL-4 mAB (11b11, BioXcell) or PBS control, as described previously [31]. Mice were also treated i.p. with 3 ml of thioglycollate alone or in combination with IL-4c on day 0 for comparison. Following sacrifice on day 4, cells were isolated from peritoneal exudate by washing the peritoneal cavity with cold PBS. Peritoneal exudate cells were treated with ACK lysis buffer (Lonza Walkersville) to lyse red blood cells and washed with PBS. Cells were either used immediately for further staining and analysis by flow cytometry or lysed with TRIzol for RNA extraction.

### Tissue preparation and histopathology

To obtain single-cell suspensions, livers were minced and digested with 100 U/ml type 8 collagenase (Sigma) and 150 µg/ml DNase I (Sigma) for 1 hour at 37°C followed by dispersal over 70 µm filters. Hepatic leukocytes were enriched by density centrifugation over a 40/80% Percoll (GE Healthcare) gradient. Spleens and MLN were dispersed over 70 µm filters, followed by lysis of splenic red blood cells with ACK lysis buffer (Invitrogen). Small intestine and colon tissue were first cleaned of mesentery, fat, and fecal contents, and then cut into ∼2 cm pieces. Tissue pieces were incubated with 1 mM DTT followed by two consecutive incubations with 30 mM EDTA and 10 mM HEPES to remove epithelial cells. The remaining intestinal tissue was then digested as described above, and leukocytes were enriched by density centrifugation over a 40/80% Percoll gradient. For histopathology, liver tissue was fixed in 10% formalin and paraffin-imbedded. Tissue sections were stained with hematoxylin and eosin for egg granuloma diameter measurements, eosinophil quantification, and scoring of microvesicular damage, as described [46], by two individuals blinded to treatment.

### *Ex vivo* stimulation

5×10^5^ cells were stimulated for 5 hours at 37°C in the presence of 10 µg/ml brefeldin A (GolgiPlug, BD Pharmingen). Phorbol 12-myristate 13-acetate (PMA, 10 ng/ml) and ionomycin (1 µg/ml) were used for polyclonal T cell stimulations. LCMV peptides GP61 and GP33 (10 µg/ml) were used for antigen-specific CD4^+^ and CD8^+^ T cell stimulations, respectively. For detection of cytokines in culture supernatants, 5×10^5^ cells were cultured for 72 hours in the presence of adult schistosome worm homogenate or schistosome egg homogenate at a protein concentration of 50 µg/ml. Cytokines were quantified using a multiplex bead-based assay (T_H_1/T_H_2/T_H_17 Cytometric Bead Array, BD Biosciences), according to the manufacturer's instructions. Samples were acquired on an LSRII with FACSDiVa software (BD Biosciences) and data were analyzed with FCAP Array software.

### Flow cytometry

#### T cell phenotyping

Cells were incubated for 30 minutes at 4°C with fluorochrome-conjugated antibodies against CD3 (500A2, BD Biosciences), CD4 (RM4-5, Invitrogen), CD8 (5H10, Invitrogen), CCR9 (CW-1.2, eBioscience), CD62L (MEL-14, eBioscience), α4β7 (DATK-32, Biolegend) and CD44 (IM7, eBioscience).

#### Intracellular cytokine staining

Following surface staining with antibodies against CD3, CD4, and CD8, cells were fixed with 2% paraformaldehyde and permeabilized with 0.5% saponin. Cells were then incubated with anti-mouse CD16/32 (eBioscience) to block Fc receptors, followed by a 30 minute incubation at 4°C with fluorochrome-conjugated antibodies against IL-4 (11B11, eBioscience), IFNγ (XMG1.2, BD Biosciences), and TNFα (MP6-XT22, eBioscience).

#### Aldefluor staining

Following surface staining for 30 minutes at 4°C with fluorochrome-conjugated antibodies against CD11b (M1/70, eBioscience), CD11c (N418, eBioscience), F4/80 (BM8, eBioscience), MMR (C068C2, Biolegend), CD80 (16-10A1, Biolegend), I-A/I-E (M5/114.15.2, BD Biosciences), Ly-6C (Al-21, BD Biosciences), Ly-6G (RB6-8C5, eBioscience) cells were washed with FACS buffer and resuspended in 300 µl of ALDEFLUOR assay buffer for further processing. Aldehyde dehydrogenase (ALDH) activity was measured using the ALDEFLUOR staining kit (StemCell Technologies) according to the manufacturer's protocol as described previously [47]–[49] with minor modifications. Briefly, cells were suspended at a concentration of 1×10^6^ cells/ml in ALDEFLUOR assay buffer containing activated ALDEFLUOR substrate, with or without the ALDH inhibitor, diethylamino-benzaldehyde (DEAB) (at a final concentration of 15 µM) and incubated at 37°C for 30 minutes. Cells were subsequently washed and resuspended in ALDEFLUOR assay buffer. Cells were kept on ice until acquisition.

#### Treg staining

Following surface staining with antibodies against CD3, CD4, CD8, and CD25 (PC61, BD Biosciences), cells were washed with PhosFlow permeabilization buffer (BD Biosciences), blocked with anti-mouse CD16/32, and stained with Foxp3 antibody (FJK-16s, eBioscience) for 1 hour at 4°C. For all experiments, dead cells were excluded with LIVE/DEAD Fixable Aqua Dead Cell Stain kit (Invitrogen). Samples were acquired on an LSRII with FACSDiVa software (BD Biosciences). Data were analyzed with FlowJo software (TreeStar).

### Cell sorting

Cells were stained with PE-conjugated anti-Siglec-F antibody (E50-2440, BD Biosciences) for 20 minutes at 4°C and then incubated with anti-PE magnetic beads (Miltenyi Biotec). Siglec-F^+^ cells were positively selected on MS columns (Miltenyi Biotec), according to the manufacturer's instructions; Siglec-F^−^ cells were collected in the flow-through. Both fractions were stained with antibodies against CD3, CD11b, and Siglec-F, and sorted directly into TRIzol (Invitrogen) using a BD FACSAria cell sorter.

### Quantitative real time (qRT)-PCR

Tissue samples were homogenized in TRIzol. RNA was collected in the aqueous extraction phase and column purified using an RNeasy kit (Qiagen). cDNA was generated using an Omniscript Reverse Transcription kit (Qiagen) with oligo-dT primers in the presence of RNasin Plus RNase inhibitor (Promega). PCR reactions were carried out with Taqman primer/probe sets (Applied Biosystems) in a StepOne Plus machine (Applied Biosystems).

### Immunofluorescence

Sections of formalin-fixed, paraffin-imbedded tissue were deparaffinized and rehydrated according to standard protocols. Slides were immersed in citrate buffer (pH 6.0) and heated in a pressure cooker for antigen retrieval. After blocking, tissue sections were stained for 1 hour at room temperature with antibodies against CD11b (M1/70, Abcam) and Raldh (Abcam) followed by a 1-hour incubation with fluorochrome-conjugated secondary antibodies. Images were acquired on a Leica DM6000B microscope.

### Intravital imaging

CX3CR1-GFP/+ mice were anesthetized with a combination of ketamine, xylazine, and acepromazine injected intraperitoneally and were kept warm on a heating pad or a pre-warmed stage. Livers of anesthetized mice were exposed by carefully cutting through the skin and peritoneum just below the rib cage and gently coaxing out a lobe of the liver. Mice were then inverted onto a pre-warmed aluminum stage insert with a 2.5 cm hole fitted with a glass coverslip secured with vacuum grease and tape. The liver was stabilized with gauze soaked in PBS to limit movement during imaging and to keep the liver moist. Mice were injected retro-orbitally with 250 µg of Hoechst 33342 to visualize nuclei and 250 µg BSA conjugated to Alexa 647 to detect blood vessels. Mice were then transferred to a heated chamber that was used to keep the microscope, objectives, mice, and stage at 37°C during imaging. Images were acquired on a Leica SP2 inverted confocal microscope with light generated from UV, 488 nm, and 633 nm laser lines and detected using tunable filters.

### Derivation and activation of bone marrow-derived macrophages

Macrophages were derived from bone marrow cells harvested from the femurs and tibias of C57BL/6 mice. Cells were differentiated for six days in the presence of fetal bovine serum (FBS) and 3T3 fibroblast supernatant containing M-CSF and cryopreserved. Thawed macrophages were rested for 12 hours, followed by activation with IL-4 (20 ng/ml) or IFNγ (50 ng/ml; all cytokines were purchased from Peprotech). Cells were lysed in TRIzol (Invitrogen) at the indicated time points for RNA extraction.

### T cell differentiation assay

4×10^5^ naïve T cells isolated from lymph nodes using the Naive CD4^+^ T Cell Isolation Kit II (Miltenyi Biotec) were cultured together with 2×10^5^ peritoneal macrophages and 1 µg/ml of soluble anti-CD3 and 5 ng/ml recombinant human IL-2 (R&D) in complete RPMI (10% FCS, 2 mM L-glutamine, 0.05 mM 2-mercaptoethanol, and 100 U of penicillin and streptomycin) for 3 d or 6 d in 12-well plates. Cultures were supplemented with fresh medium containing 5 ng/ml IL-2 on day 3. In some proliferation assays, T cells were labeled with Violet CellTracker (Invitrogen) to track cell division. On day 6, cells were stained for flow cytometry and Foxp3^+^ cells were detected by intracellular nuclear staining (see above). Under certain conditions, recombinant human TGF-β1 (R&D Systems), all-trans RA (Sigma-Aldrich), or the RA receptor inhibitor LE540 (Wako Chemicals USA) were added to culture wells.

### Statistical analysis

Statistical significance was determined with the unpaired Students's t test using Prism software (GraphPad).

### Supplemental material

Figure S1 shows that T_H_1 responses during *S. mansoni* infection are not dependent on vitamin A. Figure S2 shows that Foxp3^+^ Tregs are sustained in the thymus and small intestine during vitamin A deficiency. Figure S3 shows that LCMV-specific CD4^+^ and CD8^+^ T cell responses in the MLN are not dependent on vitamin A metabolites. Figure S4 shows that polyclonal T_H_1 responses during LCMV infection are only dependent on vitamin A in the intestinal mucosa. Figure S5 shows that retinoid-dependent expression of CCR9 on CD4^+^ T cells in the liver is not altered during infection with *S. mansoni* or LCMV. Figure S6 shows that ALDH activity and expression of Ym1 and FIZZ1 are upregulated in AAMφ induced by thioglycollate and IL-4.

## Supporting Information

Figure S1 **T_H_1 cytokine expression during** ***S. mansoni*** **infection is not retinoid-dependent.** (**A–C**) Flow cytometric analysis of intracellular cytokines expressed by cells harvested from the liver (**A**), colon (**B**), MLN, and spleen (**C**) following a 5-hour stimulation with PMA and ionomycin in the presence of brefeldin A. Results shown are gated on live CD4^+^ T cells. n = 3–5 mice per group. (**D**) Cytometric bead array analysis of cytokine concentrations in culture supernatants. MLN cells harvested from *S. mansoni*-infected (Inf) and control (Cont) mice were cultured as described in Figure 2. (**E**) qRT-PCR analysis of cytokine expression in whole MLN. Expression is normalized to HPRT. n = 3–5 mice per group. Error bars illustrate SEM. Results are representative of two (**E**) or three (**A–D**) independent experiments.
(TIF)

Figure S2 **Alterations in Foxp3^+^ regulatory T cells during vitamin A deficiency.** Flow cytometric analysis of intranuclear Foxp3 in cells harvested from the thymus and small intestine at 7 weeks (*S. mansoni*) or 7 days (LCMV) post-infection (p.i.) from A+ or A− mice. Representative contour plots are gated on live CD4^+^ T cells. n = 3–5 mice per group. Error bars illustrate SEM; #p<0.001. Results are representative of three independent experiments.
(TIF)

Figure S3 **LCMV-specific T_H_1 responses in the MLN are not vitamin A-dependent.** Flow cytometric analysis of intracellular cytokines expressed by cells harvested from the MLN of LCMV-infected mice following a 5-hour stimulation with GP61 or GP33 peptides (10 µg/mL) in the presence of brefeldin A. Representative contour plots are gated on live CD4^+^ or CD8^+^ T cells. n = 3–5 mice per group. Error bars illustrate SEM. Results are representative of three independent experiments.
(TIF)

Figure S4 **Polyclonal Th1 responses in the intestinal mucosa are retinoid-dependent.** Flow cytometric analysis of intracellular cytokines following a 5-hour stimulation with PMA and ionomycin in the presence of brefeldin A. Bars represent average frequencies of IFNγ^+^ cells within the live CD4^+^ T cell gate. n = 3–5 mice per group. Error bars illustrate SEM; *p<0.05, **p<0.01. Results are representative of three independent experiments.
(TIF)

Figure S5 **CCR9 expression by T cells in the liver is partially retinoid-dependent.** Flow cytometric analysis of cells harvested at 7 weeks (*S. mansoni*) or 7 days (LCMV) post-infection (p.i.) from A+ or A− mice. Representative contour plots are gated on live CD4+ T cells. n = 3–5 mice per group. Error bars illustrate SEM; *p<0.05, **p<0.01. Results are representative of two independent experiments.
(TIF)

Figure S6 **ALDH activity and expression of Ym1 and FIZZ1 are upregulated in AAMφ induced by thioglycollate and IL-4.** (**A**) qRT-PCR analysis of Ym1 and FIZZ1 expression in peritoneal macrophages elicited by i.p. administration of thioglycollate (TG) and/or IL-4 complexes (IL-4c) from WT and Stat6^−/−^ mice. Expression is normalized to GAPDH. n = 2–4 mice per group. Error bars illustrate SEM; Results are representative of more than three independent experiments. (**B**) Flow cytometric analysis of aldehyde dehydrogenase (ALDH) activity in peritoneal cells using the Aldefluor assay, in the presence or absence of the ALDH inhibitor, diethylaminobenzaldehyde (DEAB). Representative contour plots are gated on live F4/80^+^CD11b^+^ cells. Results are representative of more than three independent experiments.
(TIF)

## References

[1] Sommer A (2008) Vitamin a deficiency and clinical disease: an historical overview. J Nutr 138: 1835–1839. 18806089 10.1093/jn/138.10.1835

[2] Stephensen CB (2001) Vitamin A, infection, and immune function. Annu Rev Nutr 21: 167–192. 11375434 10.1146/annurev.nutr.21.1.167

[3] Hall JA, Grainger JR, Spencer SP, Belkaid Y (2011) The role of retinoic acid in tolerance and immunity. Immunity 35: 13–22. 21777796 10.1016/j.immuni.2011.07.002 PMC3418663

[4] Duester G (2000) Families of retinoid dehydrogenases regulating vitamin A function: production of visual pigment and retinoic acid. Eur J Biochem 267: 4315–4324. 10880953 10.1046/j.1432-1327.2000.01497.x

[5] Duester G, Mic FA, Molotkov A (2003) Cytosolic retinoid dehydrogenases govern ubiquitous metabolism of retinol to retinaldehyde followed by tissue-specific metabolism to retinoic acid. Chem Biol Interact 143–144: 201–210. 10.1016/s0009-2797(02)00204-1 12604205

[6] Sima A, Parisotto M, Mader S, Bhat PV (2009) Kinetic characterization of recombinant mouse retinal dehydrogenase types 3 and 4 for retinal substrates. Biochim Biophys Acta 1790: 1660–1664. 19766701 10.1016/j.bbagen.2009.09.004

[7] Iwata M, Hirakiyama A, Eshima Y, Kagechika H, Kato C, et al (2004) Retinoic acid imprints gut-homing specificity on T cells. Immunity 21: 527–538. 15485630 10.1016/j.immuni.2004.08.011

[8] Sun CM, Hall JA, Blank RB, Bouladoux N, Oukka M, et al (2007) Small intestine lamina propria dendritic cells promote de novo generation of Foxp3 T reg cells via retinoic acid. J Exp Med 204: 1775–1785. 17620362 10.1084/jem.20070602 PMC2118682

[9] Denning TL, Wang YC, Patel SR, Williams IR, Pulendran B (2007) Lamina propria macrophages and dendritic cells differentially induce regulatory and interleukin 17-producing T cell responses. Nat Immunol 8: 1086–1094. 17873879 10.1038/ni1511

[10] Coombes JL, Siddiqui KR, Arancibia-Carcamo CV, Hall J, Sun CM, et al (2007) A functionally specialized population of mucosal CD103+ DCs induces Foxp3+ regulatory T cells via a TGF-beta and retinoic acid-dependent mechanism. J Exp Med 204: 1757–1764. 17620361 10.1084/jem.20070590 PMC2118683

[11] Mora JR, von Andrian UH (2009) Role of retinoic acid in the imprinting of gut-homing IgA-secreting cells. Semin Immunol 21: 28–35. 18804386 10.1016/j.smim.2008.08.002 PMC2663412

[12] Hall JA, Cannons JL, Grainger JR, Dos Santos LM, Hand TW, et al (2011) Essential role for retinoic acid in the promotion of CD4(+) T cell effector responses via retinoic acid receptor alpha. Immunity 34: 435–447. 21419664 10.1016/j.immuni.2011.03.003 PMC3415227

[13] DePaolo RW, Abadie V, Tang F, Fehlner-Peach H, Hall JA, et al (2011) Co-adjuvant effects of retinoic acid and IL-15 induce inflammatory immunity to dietary antigens. Nature 471: 220–224. 21307853 10.1038/nature09849 PMC3076739

[14] Pino-Lagos K, Guo Y, Brown C, Alexander MP, Elgueta R, et al (2011) A retinoic acid-dependent checkpoint in the development of CD4+ T cell-mediated immunity. J Exp Med 208: 1767–1775. 21859847 10.1084/jem.20102358 PMC3171100

[15] Benson MJ, Pino-Lagos K, Rosemblatt M, Noelle RJ (2007) All-trans retinoic acid mediates enhanced T reg cell growth, differentiation, and gut homing in the face of high levels of co-stimulation. J Exp Med 204: 1765–1774. 17620363 10.1084/jem.20070719 PMC2118687

[16] Mucida D, Park Y, Kim G, Turovskaya O, Scott I, et al (2007) Reciprocal TH17 and regulatory T cell differentiation mediated by retinoic acid. Science 317: 256–260. 17569825 10.1126/science.1145697

[17] Iwata M, Eshima Y, Kagechika H (2003) Retinoic acids exert direct effects on T cells to suppress Th1 development and enhance Th2 development via retinoic acid receptors. Int Immunol 15: 1017–1025. 12882839 10.1093/intimm/dxg101

[18] Stephensen CB, Rasooly R, Jiang X, Ceddia MA, Weaver CT, et al (2002) Vitamin A enhances in vitro Th2 development via retinoid X receptor pathway. J Immunol 168: 4495–4503. 11970994 10.4049/jimmunol.168.9.4495

[19] Dawson HD, Collins G, Pyle R, Key M, Weeraratna A, et al (2006) Direct and indirect effects of retinoic acid on human Th2 cytokine and chemokine expression by human T lymphocytes. BMC Immunol 7: 27. 17118196 10.1186/1471-2172-7-27 PMC1665462

[20] Schuster GU, Kenyon NJ, Stephensen CB (2008) Vitamin A deficiency decreases and high dietary vitamin A increases disease severity in the mouse model of asthma. J Immunol 180: 1834–1842. 18209081 10.4049/jimmunol.180.3.1834

[21] Carman JA, Pond L, Nashold F, Wassom DL, Hayes CE (1992) Immunity to Trichinella spiralis infection in vitamin A-deficient mice. J Exp Med 175: 111–120. 1730911 10.1084/jem.175.1.111 PMC2119062

[22] Cantorna MT, Nashold FE, Hayes CE (1994) In vitamin A deficiency multiple mechanisms establish a regulatory T helper cell imbalance with excess Th1 and insufficient Th2 function. J Immunol 152: 1515–1522. 8120366

[23] Anthony RM, Rutitzky LI, Urban JF Jr, Stadecker MJ, Gause WC (2007) Protective immune mechanisms in helminth infection. Nat Rev Immunol 7: 975–987. 18007680 10.1038/nri2199 PMC2258092

[24] Allen JE, Maizels RM (2011) Diversity and dialogue in immunity to helminths. Nat Rev Immunol 11: 375–388. 21610741 10.1038/nri2992

[25] Pearce EJ, MacDonald AS (2002) The immunobiology of schistosomiasis. Nat Rev Immunol 2: 499–511. 12094224 10.1038/nri843

[26] WHO (2009) Global prevalence of vitamin A deficiency in populations at risk 1995–2005. Geneva: World Health Organization.

[27] Kaplan MH, Whitfield JR, Boros DL, Grusby MJ (1998) Th2 cells are required for the Schistosoma mansoni egg-induced granulomatous response. J Immunol 160: 1850–1856. 9469446

[28] Brunet LR, Finkelman FD, Cheever AW, Kopf MA, Pearce EJ (1997) IL-4 protects against TNF-alpha-mediated cachexia and death during acute schistosomiasis. J Immunol 159: 777–785. 9218595

[29] King IL, Mohrs M (2009) IL-4-producing CD4+ T cells in reactive lymph nodes during helminth infection are T follicular helper cells. J Exp Med 206: 1001–1007. 19380638 10.1084/jem.20090313 PMC2715031

[30] Zaretsky AG, Taylor JJ, King IL, Marshall FA, Mohrs M, et al (2009) T follicular helper cells differentiate from Th2 cells in response to helminth antigens. J Exp Med 206: 991–999. 19380637 10.1084/jem.20090303 PMC2715032

[31] Jenkins SJ, Ruckerl D, Cook PC, Jones LH, Finkelman FD, et al (2011) Local macrophage proliferation, rather than recruitment from the blood, is a signature of TH2 inflammation. Science 332: 1284–1288. 21566158 10.1126/science.1204351 PMC3128495

[32] Geissmann F, Jung S, Littman DR (2003) Blood monocytes consist of two principal subsets with distinct migratory properties. Immunity 19: 71–82. 12871640 10.1016/s1074-7613(03)00174-2

[33] Loke P, MacDonald AS, Robb A, Maizels RM, Allen JE (2000) Alternatively activated macrophages induced by nematode infection inhibit proliferation via cell-to-cell contact. Eur J Immunol 30: 2669–2678. 11009101 10.1002/1521-4141(200009)30:9<2669::AID-IMMU2669>3.0.CO;2-1

[34] Pesce JT, Ramalingam TR, Mentink-Kane MM, Wilson MS, El Kasmi KC, et al (2009) Arginase-1-expressing macrophages suppress Th2 cytokine-driven inflammation and fibrosis. PLoS Pathog 5: e1000371. 19360123 10.1371/journal.ppat.1000371 PMC2660425

[35] Gordon S, Martinez FO (2010) Alternative activation of macrophages: mechanism and functions. Immunity 32: 593–604. 20510870 10.1016/j.immuni.2010.05.007

[36] Elgueta R, Sepulveda FE, Vilches F, Vargas L, Mora JR, et al (2008) Imprinting of CCR9 on CD4 T cells requires IL-4 signaling on mesenteric lymph node dendritic cells. J Immunol 180: 6501–6507. 18453568 10.4049/jimmunol.180.10.6501

[37] Yokota A, Takeuchi H, Maeda N, Ohoka Y, Kato C, et al (2009) GM-CSF and IL-4 synergistically trigger dendritic cells to acquire retinoic acid-producing capacity. Int Immunol 21: 361–377. 19190084 10.1093/intimm/dxp003 PMC2660862

[38] Manicassamy S, Reizis B, Ravindran R, Nakaya H, Salazar-Gonzalez RM, et al (2010) Activation of beta-catenin in dendritic cells regulates immunity versus tolerance in the intestine. Science 329: 849–853. 20705860 10.1126/science.1188510 PMC3732486

[39] Manicassamy S, Ravindran R, Deng J, Oluoch H, Denning TL, et al (2009) Toll-like receptor 2-dependent induction of vitamin A-metabolizing enzymes in dendritic cells promotes T regulatory responses and inhibits autoimmunity. Nat Med 15: 401–409. 19252500 10.1038/nm.1925 PMC2768543

[40] Szatmari I, Pap A, Ruhl R, Ma JX, Illarionov PA, et al (2006) PPARgamma controls CD1d expression by turning on retinoic acid synthesis in developing human dendritic cells. J Exp Med 203: 2351–2362. 16982809 10.1084/jem.20060141 PMC2118109

[41] Stock A, Booth S, Cerundolo V (2011) Prostaglandin E2 suppresses the differentiation of retinoic acid-producing dendritic cells in mice and humans. J Exp Med 208: 761–773. 21444662 10.1084/jem.20101967 PMC3135350

[42] Molenaar R, Knippenberg M, Goverse G, Olivier BJ, de Vos AF, et al (2011) Expression of retinaldehyde dehydrogenase enzymes in mucosal dendritic cells and gut-draining lymph node stromal cells is controlled by dietary vitamin A. J Immunol 186: 1934–1942. 21220692 10.4049/jimmunol.1001672

[43] Feng T, Cong Y, Qin H, Benveniste EN, Elson CO (2010) Generation of mucosal dendritic cells from bone marrow reveals a critical role of retinoic acid. J Immunol 185: 5915–5925. 20944006 10.4049/jimmunol.1001233 PMC4454342

[44] Ueki S, Mahemuti G, Oyamada H, Kato H, Kihara J, et al (2008) Retinoic acids are potent inhibitors of spontaneous human eosinophil apoptosis. J Immunol 181: 7689–7698. 19017957 10.4049/jimmunol.181.11.7689

[45] Wada Y, Hisamatsu T, Kamada N, Okamoto S, Hibi T (2009) Retinoic acid contributes to the induction of IL-12-hypoproducing dendritic cells. Inflamm Bowel Dis 15: 1548–1556. 19340880 10.1002/ibd.20934

[46] Wang X, Allen C, Ballow M (2007) Retinoic acid enhances the production of IL-10 while reducing the synthesis of IL-12 and TNF-alpha from LPS-stimulated monocytes/macrophages. J Clin Immunol 27: 193–200. 17253143 10.1007/s10875-006-9068-5

[47] Spiegl N, Didichenko S, McCaffery P, Langen H, Dahinden CA (2008) Human basophils activated by mast cell-derived IL-3 express retinaldehyde dehydrogenase-II and produce the immunoregulatory mediator retinoic acid. Blood 112: 3762–3771. 18495959 10.1182/blood-2008-01-135251

[48] Cook PC, Jones LH, Jenkins SJ, Wynn TA, Allen JE, et al (2012) Alternatively activated dendritic cells regulate CD4+ T-cell polarization in vitro and in vivo. Proc Natl Acad Sci U S A 109: 9977–9982. 22660926 10.1073/pnas.1121231109 PMC3382483

[49] Hill JA, Hall JA, Sun CM, Cai Q, Ghyselinck N, et al (2008) Retinoic acid enhances Foxp3 induction indirectly by relieving inhibition from CD4+CD44hi Cells. Immunity 29: 758–770. 19006694 10.1016/j.immuni.2008.09.018 PMC3140207

[50] Kreider T, Anthony RM, Urban JF Jr, Gause WC (2007) Alternatively activated macrophages in helminth infections. Curr Opin Immunol 19: 448–453. 17702561 10.1016/j.coi.2007.07.002 PMC2000338

[51] Allen JE, Wynn TA (2011) Evolution of Th2 immunity: a rapid repair response to tissue destructive pathogens. PLoS Pathog 7: e1002003. 21589896 10.1371/journal.ppat.1002003 PMC3093361

[52] Herbert DR, Holscher C, Mohrs M, Arendse B, Schwegmann A, et al (2004) Alternative macrophage activation is essential for survival during schistosomiasis and downmodulates T helper 1 responses and immunopathology. Immunity 20: 623–635. 15142530 10.1016/s1074-7613(04)00107-4

[53] Hotez PJ, Bethony JM, Diemert DJ, Pearson M, Loukas A (2010) Developing vaccines to combat hookworm infection and intestinal schistosomiasis. Nat Rev Microbiol 8: 814–826. 20948553 10.1038/nrmicro2438

