# Supplementary figures and images for: Upregulation of Retinal Dehydrogenase 2 in Alternatively Activated Macrophages during Retinoid-dependent Type-2 Immunity to Helminth Infection in Mice
Source: PLoS Pathog. 2012 Aug 23;8(8):e1002883. doi: 10.1371/journal.ppat.1002883 (PMC3426520; doi:10.1371/journal.ppat.1002883)

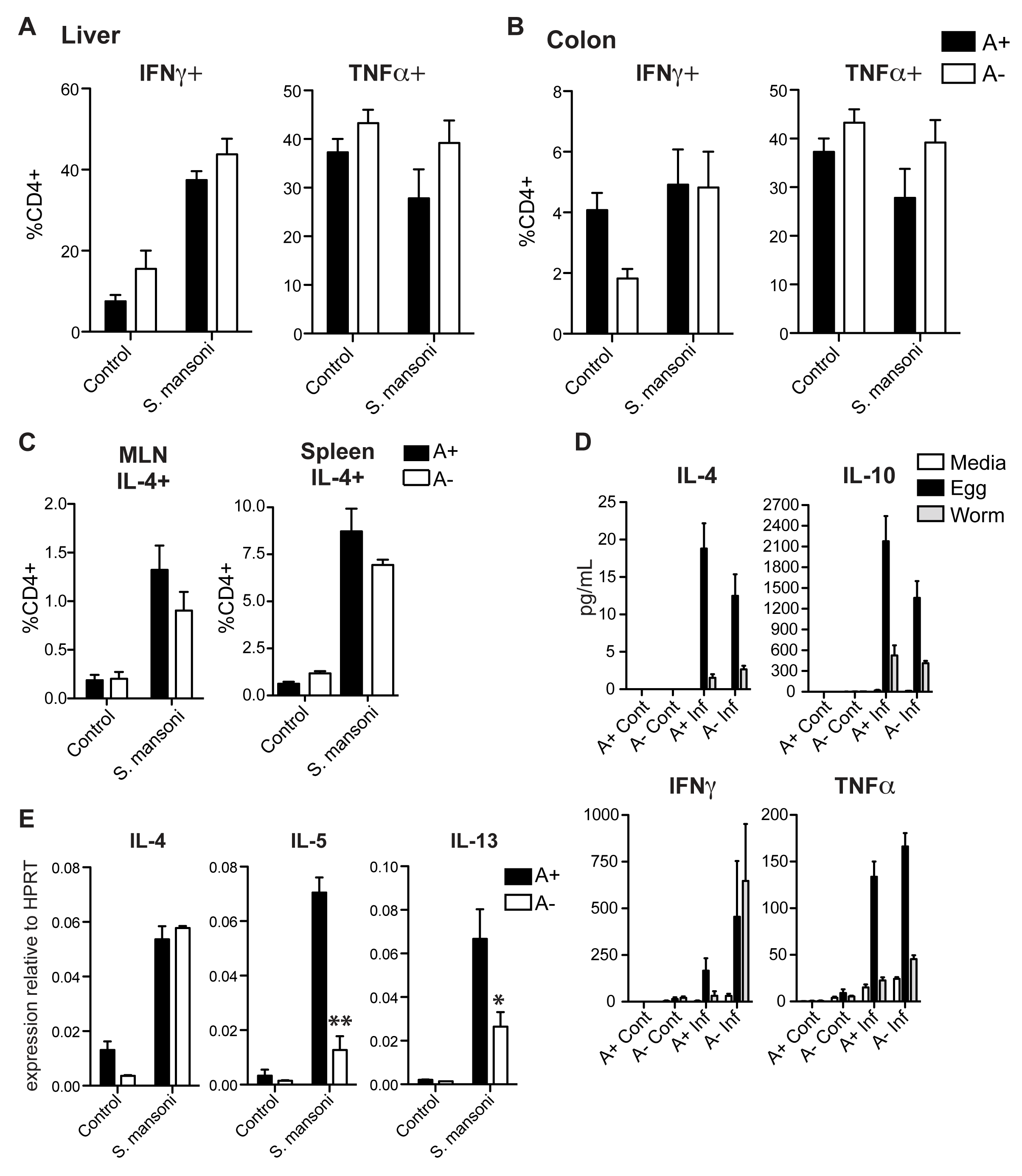

Supplement: Figure S1 — TH1 cytokine expression during S. mansoni infection is not retinoid-dependent. (A–C) Flow cytometric analysis of intracellular cytokines expressed by cells harvested from the liver (A), colon (B), MLN, and spleen (C) following a 5-hour stimulation with PMA and ionomycin in the presence of brefeldin A. Results shown are gated on live CD4+ T cells. n = 3–5 mice per group. (D) Cytometric bead array analysis of cytokine concentrations in culture supernatants. MLN cells harvested from S. mansoni-infected (Inf) and control (Cont) mice were cultured as described in Figure 2. (E) qRT-PCR analysis of cytokine expression in whole MLN. Expression is normalized to HPRT. n = 3–5 mice per group. Error bars illustrate SEM. Results are representative of two (E) or three (A–D) independent experiments. (TIF) [file ppat.1002883.s001.tif]

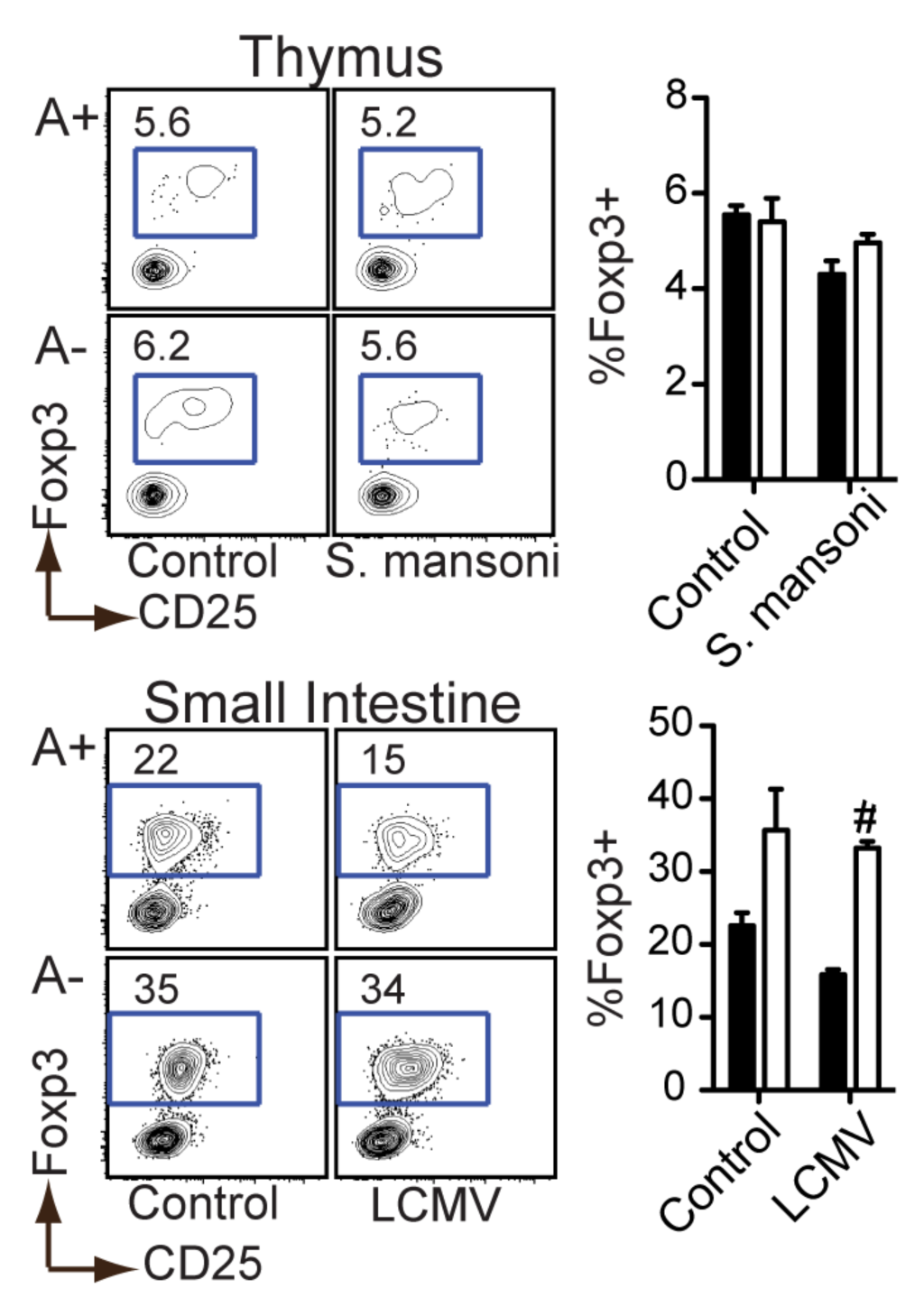

Supplement: Figure S2 — Alterations in Foxp3+ regulatory T cells during vitamin A deficiency. Flow cytometric analysis of intranuclear Foxp3 in cells harvested from the thymus and small intestine at 7 weeks (S. mansoni) or 7 days (LCMV) post-infection (p.i.) from A+ or A− mice. Representative contour plots are gated on live CD4+ T cells. n = 3–5 mice per group. Error bars illustrate SEM; #p<0.001. Results are representative of three independent experiments. (TIF) [file ppat.1002883.s002.tif]

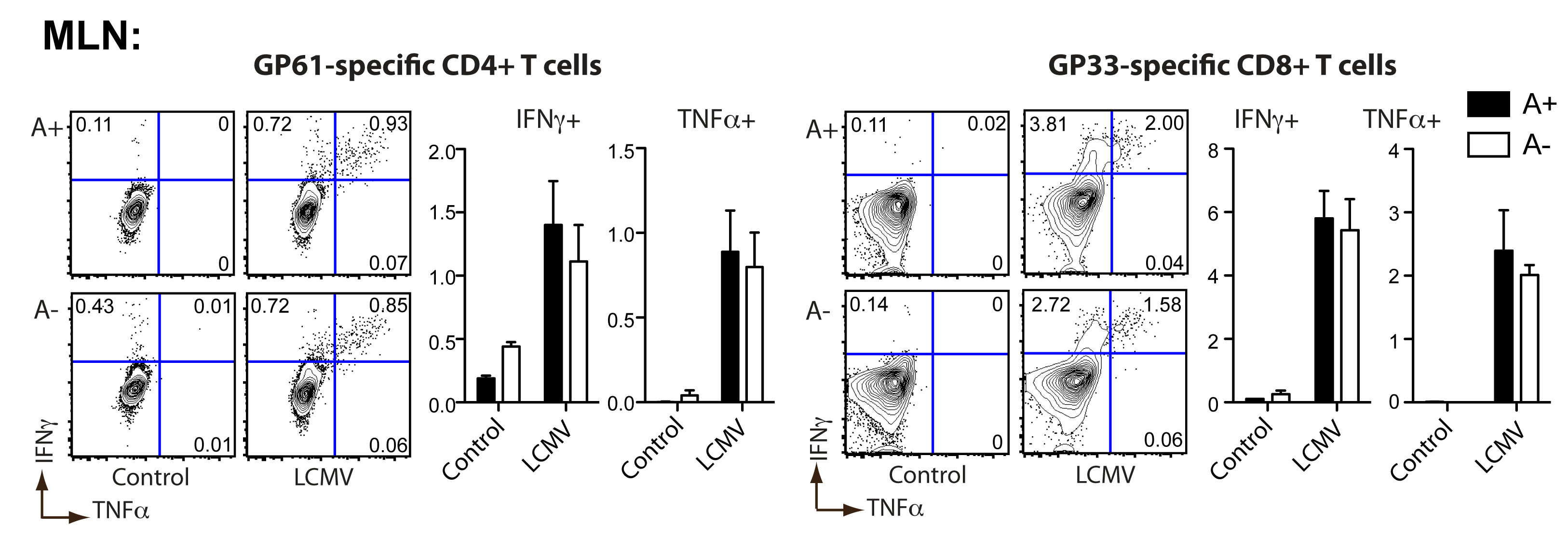

Supplement: Figure S3 — LCMV-specific TH1 responses in the MLN are not vitamin A-dependent. Flow cytometric analysis of intracellular cytokines expressed by cells harvested from the MLN of LCMV-infected mice following a 5-hour stimulation with GP61 or GP33 peptides (10 µg/mL) in the presence of brefeldin A. Representative contour plots are gated on live CD4+ or CD8+ T cells. n = 3–5 mice per group. Error bars illustrate SEM. Results are representative of three independent experiments. (TIF) [file ppat.1002883.s003.tif]

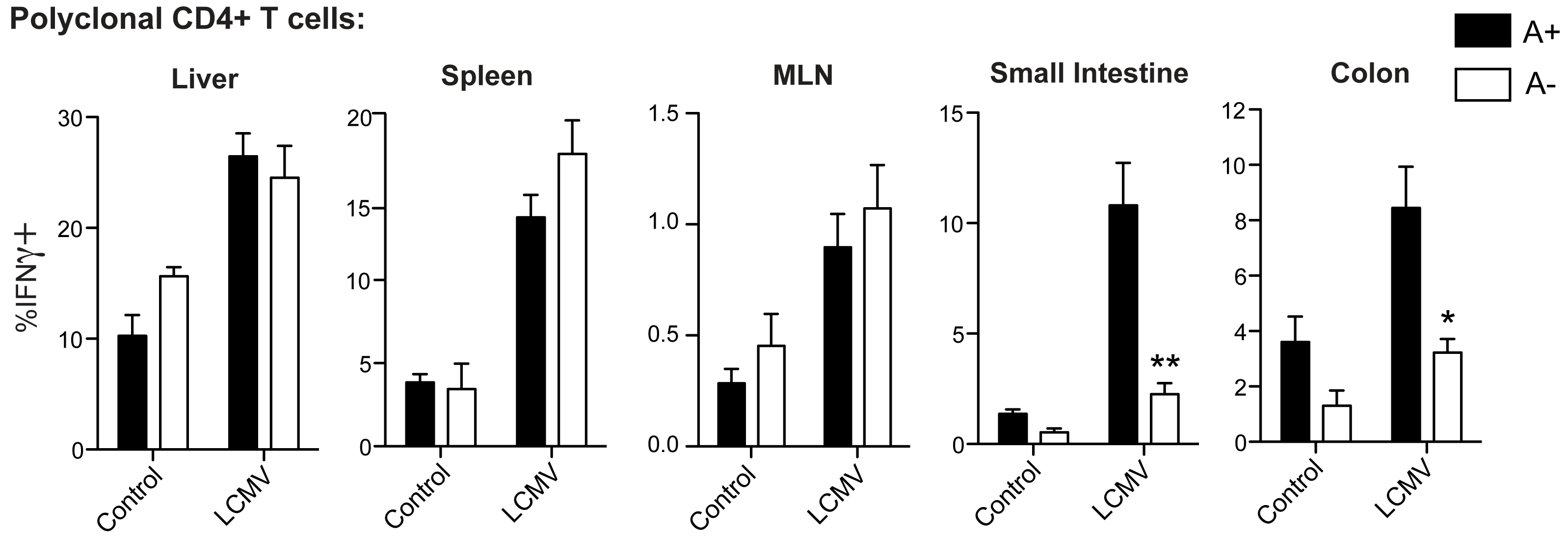

Supplement: Figure S4 — Polyclonal Th1 responses in the intestinal mucosa are retinoid-dependent. Flow cytometric analysis of intracellular cytokines following a 5-hour stimulation with PMA and ionomycin in the presence of brefeldin A. Bars represent average frequencies of IFNγ+ cells within the live CD4+ T cell gate. n = 3–5 mice per group. Error bars illustrate SEM; *p<0.05, **p<0.01. Results are representative of three independent experiments. (TIF) [file ppat.1002883.s004.tif]

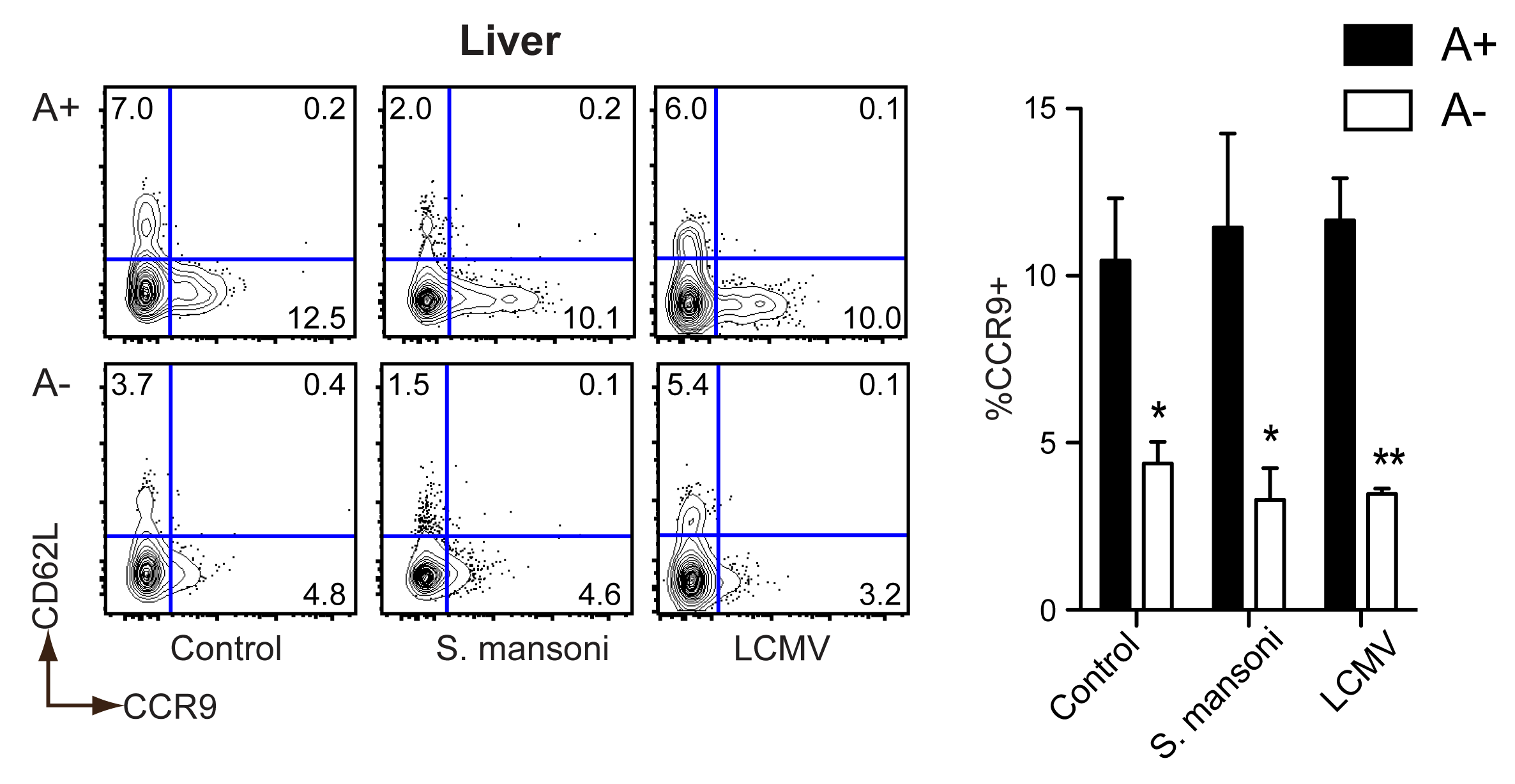

Supplement: Figure S5 — CCR9 expression by T cells in the liver is partially retinoid-dependent. Flow cytometric analysis of cells harvested at 7 weeks (S. mansoni) or 7 days (LCMV) post-infection (p.i.) from A+ or A− mice. Representative contour plots are gated on live CD4+ T cells. n = 3–5 mice per group. Error bars illustrate SEM; *p<0.05, **p<0.01. Results are representative of two independent experiments. (TIF) [file ppat.1002883.s005.tif]

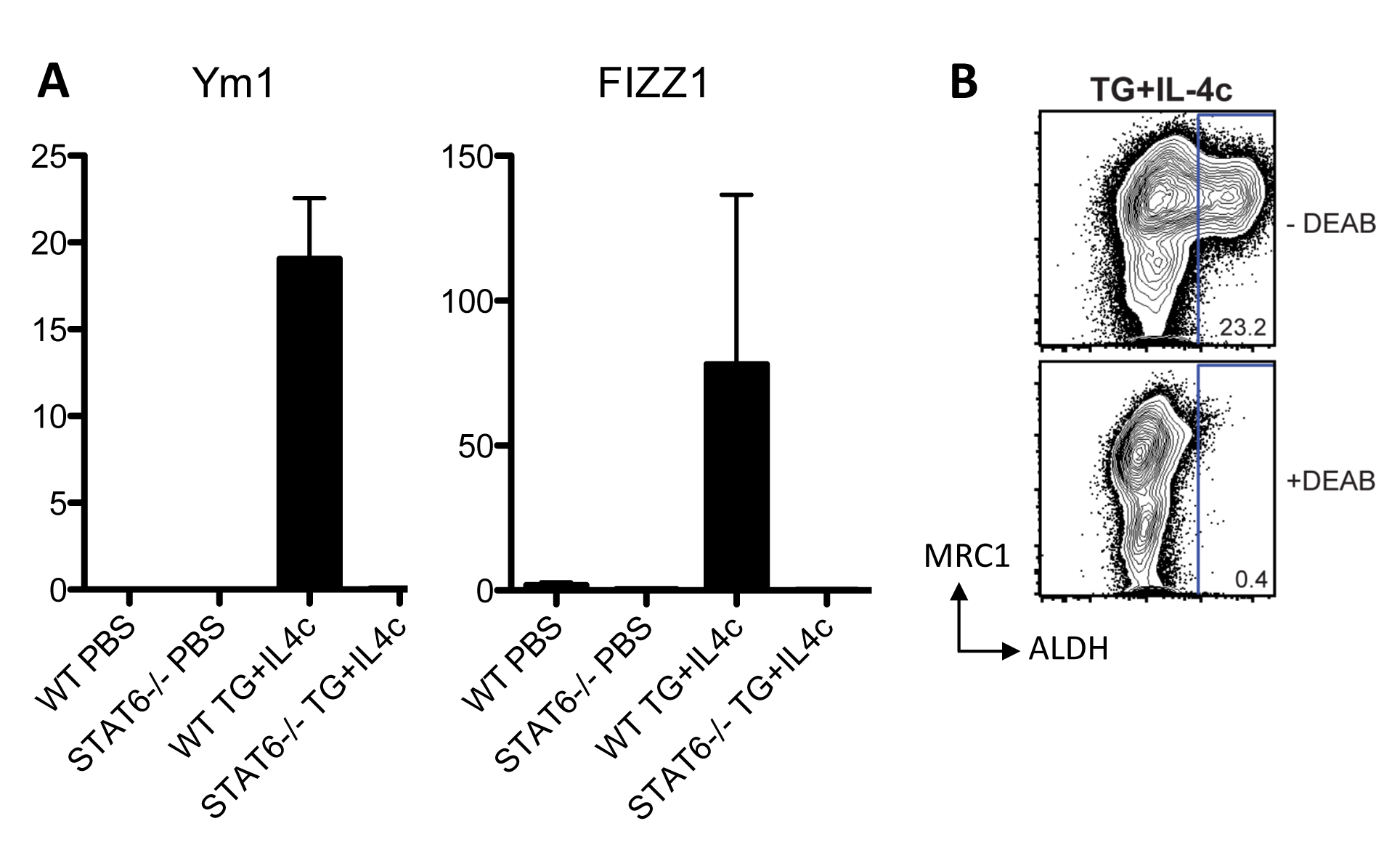

Supplement: Figure S6 — ALDH activity and expression of Ym1 and FIZZ1 are upregulated in AAMφ induced by thioglycollate and IL-4. (A) qRT-PCR analysis of Ym1 and FIZZ1 expression in peritoneal macrophages elicited by i.p. administration of thioglycollate (TG) and/or IL-4 complexes (IL-4c) from WT and Stat6−/− mice. Expression is normalized to GAPDH. n = 2–4 mice per group. Error bars illustrate SEM; Results are representative of more than three independent experiments. (B) Flow cytometric analysis of aldehyde dehydrogenase (ALDH) activity in peritoneal cells using the Aldefluor assay, in the presence or absence of the ALDH inhibitor, diethylaminobenzaldehyde (DEAB). Representative contour plots are gated on live F4/80+CD11b+ cells. Results are representative of more than three independent experiments. (TIF) [file ppat.1002883.s006.tif]
